# How Rigidity and Conjugation of Bidentate Ligands Affect the Geometry and Photophysics of Iron *N*-Heterocyclic Complexes: A Comparative Study

**DOI:** 10.1021/acs.inorgchem.3c03972

**Published:** 2024-02-29

**Authors:** Om Prakash, Pavel Chábera, Nidhi Kaul, Valtýr F. Hlynsson, Nils W. Rosemann, Iria Bolaño Losada, Yen Tran Hoang Hai, Ping Huang, Jesper Bendix, Tore Ericsson, Lennart Häggström, Arvind Kumar Gupta, Daniel Strand, Arkady Yartsev, Reiner Lomoth, Petter Persson, Kenneth Wärnmark

**Affiliations:** ‡ Centre for Analysis and Synthesis, Department of Chemistry, Lund University, Box 124, SE-22100 Lund, Sweden; § Chemical Physics Division, Department of Chemistry, Lund University, Box 124, SE-22100 Lund, Sweden; ⊥ Department of Chemistry − Ångström Laboratory, Uppsala University, Box 523, SE-751 20 Uppsala, Sweden; ∥ Theoretical Chemistry Division, Department of Chemistry, Lund University, Box 124, SE-22100 Lund, Sweden; ¶ Department of Chemistry, University of Copenhagen, Universitetsparken 5, DK-2100 Copenhagen, Denmark; # Department of Physics − Ångström Laboratory, Uppsala University, Box 523, SE-751 20 Uppsala, Sweden

## Abstract

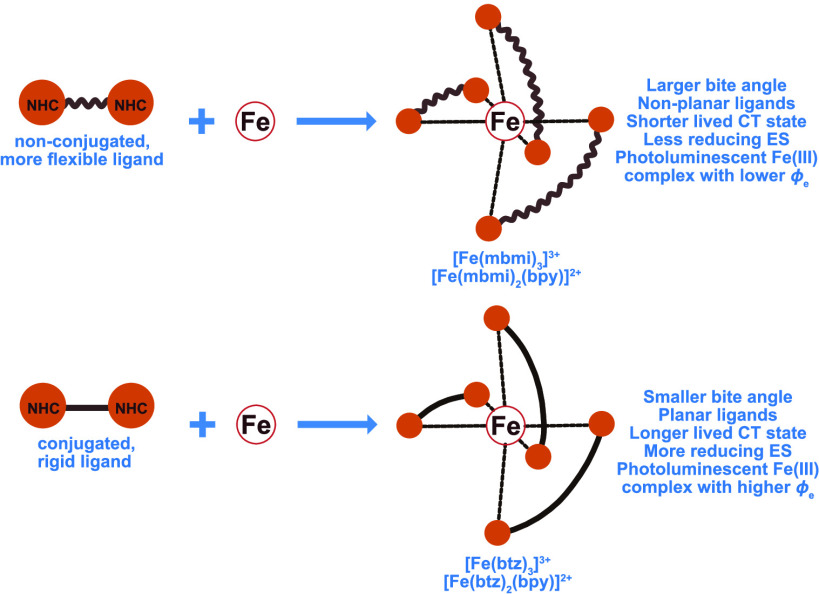

Two iron complexes featuring the bidentate, nonconjugated N-heterocyclic carbene (NHC) 1,1′-methylenebis(3-methylimidazol-2-ylidene) (mbmi) ligand, where the two NHC moieties are separated by a methylene bridge, have been synthesized to exploit the combined influence of geometric and electronic effects on the ground- and excited-state properties of homoleptic Fe^III^-hexa-NHC [Fe(mbmi)_3_](PF_6_)_3_ and heteroleptic Fe^II^-tetra-NHC [Fe(mbmi)_2_(bpy)](PF_6_)_2_ (bpy = 2,2′-bipyridine) complexes. They are compared to the reported Fe^III^-hexa-NHC [Fe(btz)_3_](PF_6_)_3_ and Fe^II^-tetra-NHC [Fe(btz)_2_(bpy)](PF_6_)_2_ complexes containing the conjugated, bidentate mesoionic NHC ligand 3,3′-dimethyl-1,1′-bis(*p*-tolyl)-4,4′-bis(1,2,3-triazol-5-ylidene) (btz). The observed geometries of [Fe(mbmi)_3_](PF_6_)_3_ and [Fe(mbmi)_2_(bpy)](PF_6_)_2_ are evaluated through L–Fe–L bond angles and ligand planarity and compared to those of [Fe(btz)_3_](PF_6_)_3_ and [Fe(btz)_2_(bpy)](PF_6_)_2_. The Fe^II^/Fe^III^ redox couples of [Fe(mbmi)_3_](PF_6_)_3_ (−0.38 V) and [Fe(mbmi)_2_(bpy)](PF_6_)_2_ (−0.057 V, both vs Fc^+/0^) are less reducing than [Fe(btz)_3_](PF_6_)_3_ and [Fe(btz)_2_(bpy)](PF_6_)_2_. The two complexes show intense absorption bands in the visible region: [Fe(mbmi)_3_](PF_6_)_3_ at 502 nm (ligand-to-metal charge transfer, ^2^LMCT) and [Fe(mbmi)_2_(bpy)](PF_6_)_2_ at 410 and 616 nm (metal-to-ligand charge transfer, ^3^MLCT). Lifetimes of 57.3 ps (^2^LMCT) for [Fe(mbmi)_3_](PF_6_)_3_ and 7.6 ps (^3^MLCT) for [Fe(mbmi)_2_(bpy)](PF_6_)_2_ were probed and are somewhat shorter than those for [Fe(btz)_3_](PF_6_)_3_ and [Fe(btz)_2_(bpy)](PF_6_)_2_. [Fe(mbmi)_3_](PF_6_)_3_ exhibits photoluminescence at 686 nm (^2^LMCT) in acetonitrile at room temperature with a quantum yield of (1.2 ± 0.1) × 10^–4^, compared to (3 ± 0.5) × 10^–4^ for [Fe(btz)_3_](PF_6_)_3_.

## Abstract 2

Two new iron N-heterocyclic (NHC) complexes, [Fe(III)(mbmi)_3_](PF_6_)_3_ and [Fe(II)(mbmi)_2_(bpy)](PF_6_) (top), have been synthesized where the NHC moieties are electronically nonconjugated and more flexible. Their electrochemical and photophysical properties were compared to the two similar reference iron NHC complexes, [Fe^III^(btz)_3_](PF_6_)_3_ and [Fe^II^(btz)_2_(bpy)](PF_6_)_2_ (bottom), where the NHC moieties are electronically conjugated.

## Introduction

Molecular photosensitizers based on earth-abundant transition-metal complexes have been emerging rapidly in recent years, presenting promising opportunities to develop sustainable-energy devices for solar-energy harvesting.^1−10^ Transition-metal complexes based on noble metals such as ruthenium, osmium, and iridium are established, efficient photosensitizers due to their, typically, long-lived charge-transfer (CT) excited states and tunability of many photophysical properties.^11−16^ However, their utilization on a large scale has been limited due to the scarcity, expense, and sometimes toxic nature of the metal.^17^ The low cost and abundance as well as the relatively nontoxic properties of first-row transition metals, such as iron, make them attractive candidates to replace noble metals for the development of large-scale light-harvesting devices.^18−20^ The electronic structure of iron(II) polypyridine complexes is inherently different from that of their ruthenium(II) analogues, making them far less useful in photochemical applications.^21^ For instance, they generally suffer from very short lifetimes (<150 fs) of the metal-to-ligand charge-transfer (MLCT) states, caused by the fast nonradiative deactivation through their lower-energy metal-centered (MC) states.^22−26^ The strong σ donation of *N*-heterocyclic carbene (NHC) ligands in iron(II) complexes, sometimes in combination with π acceptance of pyridine moieties, can effectively be utilized to tune the energy levels of the excited-state manifold and has led to significant improvements in the MLCT lifetimes, now approaching the nanosecond excited-state milestone for Fe-NHC complexes,^27−35^ with the complex [Fe^II^(btz)_3_](PF_6_)_3_ [btz = 3,3′-dimethyl-1,1′-bis(*p*-tolyl)-4,4′-bis(1,2,3-triazole-5-ylidene)] containing strongly σ-donating mesoionic moieties being a privileged structure with an ^3^MLCT lifetime of 0.5 ns^30^ and demonstrated as a catalytically active intermediate in photocatalytic redox chemistry.^36,37^ Compared to the traditional NHC moieties, the mesoionic carbenes (MICs) are even more strongly σ-donating compared to normal NHC moieties due to the formal negative charge on the carbene carbon in the classical drawings of the resonance structure.^30^ Alternative strategies, such as exchanging the pyridine moiety for carbanions in an Fe-NHC complex and using a ligand based on benzannulated phenanthridine and quinoline heterocycles paired with amido donors, as introduced by Herbert et al.,^38^ or a ligand based on phenanthroline containing a carbanionic phenyl donor, as introduced by Berkefeld et al.,^39^ have achieved MLCT lifetimes of iron(II) complexes just above the nanosecond threshold. Important breakthroughs to prolongation of the ligand-to-metal charge-transfer (LMCT) state lifetimes of two iron(III) complexes have been made by introducing six NHC moieties to the iron center, achieving LMCT lifetimes of 100 ps ([Fe^III^(btz)_3_](PF_6_)_3_)^40^ and 2 ns {[Fe^III^(phtmeimb)_2_]^+^ (phtmeimb = [phenyltris(3-methylimidazolin-2-ylidene)borate^–^])}.^41^ Interestingly, those were the first iron complexes shown to exhibit photoluminescence at room temperature.^42^ After the successes with strategic ligand design as shown above, we were interested in investigating further the influence of the ligand environment of iron complexes on their photophysical properties. One such approach is to, through ligand design, reduce the strain around the metal center upon coordination, inviting more ideal octahedral geometry and thus increasing the ligand-field splitting.^43−47^ Another approach is to insert one or more sp^3^-hybridized carbon atoms between the NHC moieties of the ligand, breaking the conjugation between them.^48−51^ Until very recently, all iron(II/III) complexes showing advantageous photophysical properties included bidentate and meridional, tridentate NHC/pyridine ligands where the NHC motifs are in electronic conjugation;^27−31,33−35,52−58^ then Gros and co-workers reported nonconjugated tridentate Fe^II^-NHC complexes and discussed the effect of structural flexibility on their excited-state properties.^59^ Since then, nonconjugated, bidentate NHC ligands have shown the structural influence that their iron(II) and iron(III) complexes can have on catalytic processes.^60−62^ However, there are no examples of functional Fe-NHC photosensitizers having bidentate ligands employing this design motif to date.

**Figure 1:**
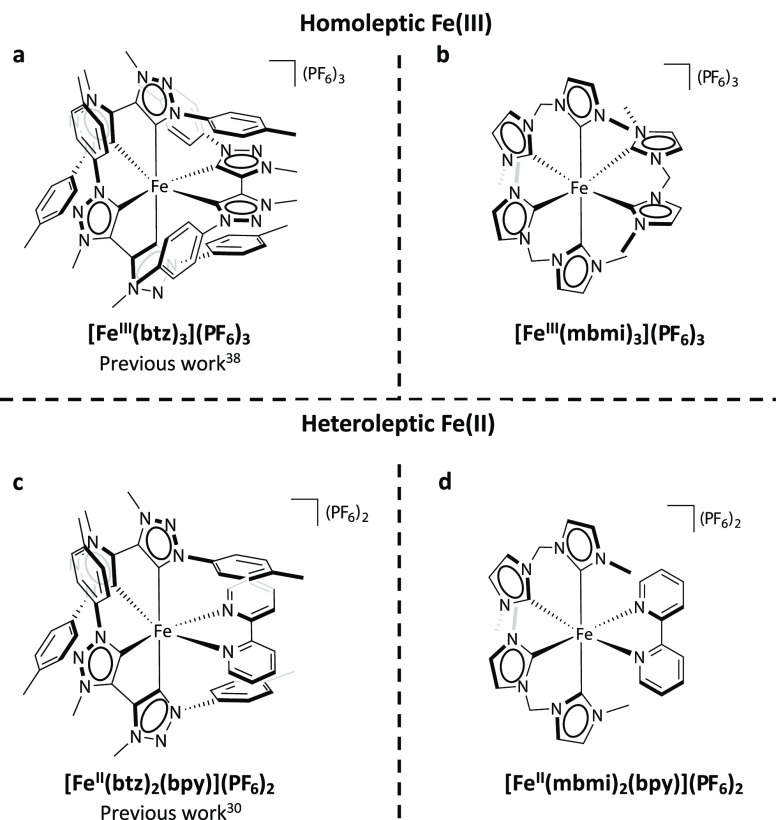
Chemical structures of the complexes compared in the study. Homoleptic [Fe^III^(mbmi)_3_](PF_6_)_3_ (b) is compared to the previously reported [Fe^III^(btz)_3_](PF_6_)_3_ (a) and heteroleptic [Fe^II^(mbmi)_2_(bpy)](PF_6_)_2_ (d) to [Fe^II^(btz)_2_(bpy)](PF_6_)_2_ (c).

Herein, we report the design and syntheses of homoleptic, hexa-NHC [Fe^III^(mbmi)_3_](PF_6_)_3_ (mbmi = 1,1′-methylenebis(3-methylimidazol-2-ylidene) and heteroleptic, tetra-NHC [Fe^II^(mbmi)_2_(bpy)](PF_6_)_2_ (bpy = 2,2′-bipyridine) complexes based on the nonconjugated [mbmiH_2_](PF_6_)_2_ (mbmiH_2_ = [1,1′-methylenebis(3-methyl-1*H*-imidazolium)]^2+^) NHC ligand precursor as introduced by Braband et al.,^63^ prompted by the prospect of exploiting how the ground- and excited-state geometries and electronic properties change when the conjugation is broken between two NHC moieties in the ligand and thus introducing more flexibility in the adapted geometry of the ligand. The properties of the complexes reported here were compared to the previously reported complexes homoleptic, hexa-NHC [Fe^III^(btz)_3_](PF_6_)_3_^40^ and heteroleptic, tetra-NHC [Fe^II^(btz)_2_(bpy)](PF_6_)_2_,^30^ respectively (Figure 1). These feature the bidentate, conjugated, MIC ligand btz and allow for a geometric and electronic comparison to [Fe^III^(mbmi)_3_](PF_6_)_3_ and [Fe^II^(mbmi)_2_(bpy)](PF_6_)_2_. Although the two sets of bidentate NHC ligands (mbmi and btz) involved in this study feature different types of NHC moieties, the influence of the ligand nature on the excited-state properties of their respective complexes is usually smaller than that of other variables, such as the number of NHC moieties and their geometrical positions.^27−35^ A structural comparison between complexes with different numbers of NHC moieties, across two NHC types, will improve the understanding of how different underlying factors affect the properties of Fe-NHC complexes.

## Results and Discussion

### Synthesis and Characterization

**Scheme 1:**
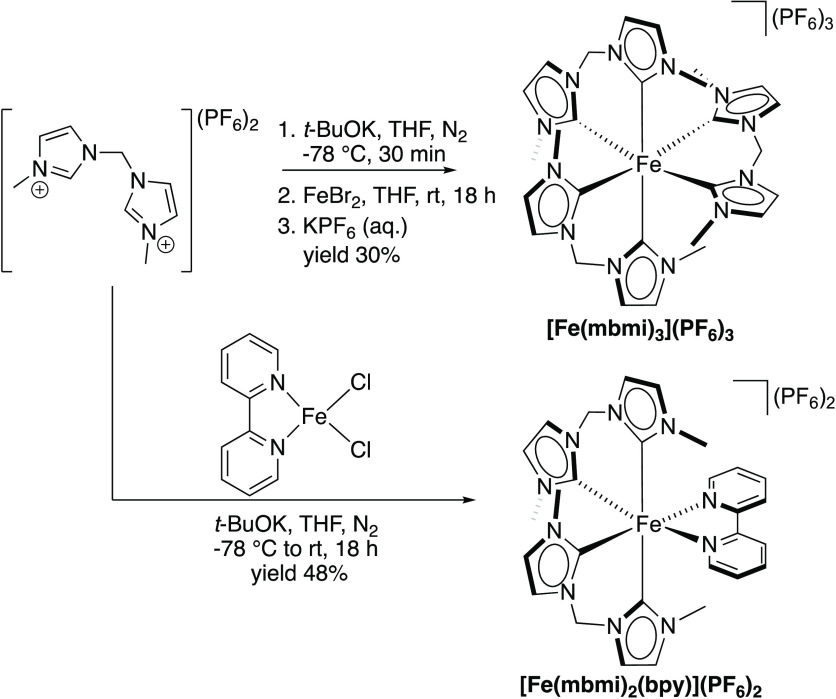
Syntheses of Homoleptic [Fe^III^(mbmi)_3_](PF_6_)_3_ and Heteroleptic [Fe^II^(mbmi)_2_(bpy)](PF_6_)_2_

The homoleptic [Fe(mbmi)_3_](PF_6_)_3_ and heteroleptic [Fe(mbmi)_2_(bpy)](PF_6_)_2_ complexes were synthesized by methods based on previously established protocols (Figure 1 and Scheme 1).^27−31,33−35^ The free carbenes of [mbmiH_2_](PF_6_)_2_^63^ were generated in situ using potassium *tert*-butoxide (*t*-BuOK) at −78 °C under a N_2_ atmosphere. The free carbene was reacted with an appropriate iron precursor, anhydrous FeBr_2_ or [Fe(bpy)Cl_2_],^64^ yielding [Fe(mbmi)_3_](PF_6_)_3_ and [Fe(mbmi)_2_(bpy)](PF_6_)_2_, respectively. The two complexes were purified with size-exclusion chromatography, followed by recrystallization to give dark red and green crystals, respectively, and their identities and purities were established through NMR spectroscopy, CHN elemental analysis, high-resolution mass spectrometry (HR-MS), and single-crystal X-ray diffraction (scXRD) analyses.

Despite being paramagnetic, the hexa-NHC complex [Fe^III^(mbmi)_3_](PF_6_)_3_ interestingly shows a well-resolved ^1^H NMR spectrum, an observation previously made for other hexa-NHC iron(III) complexes.^41,65^ As expected, the diamagnetic tetra-NHC complex [Fe^II^(mbmi)_2_(bpy)](PF_6_)_2_ shows well-resolved and characteristic ^1^H and ^13^C NMR spectra. Both complexes are stable in the solid state as well as in an acetonitrile solution for several days at ambient temperature when exposed to air and moisture.

### Structural Discussions

**Figure 2:**
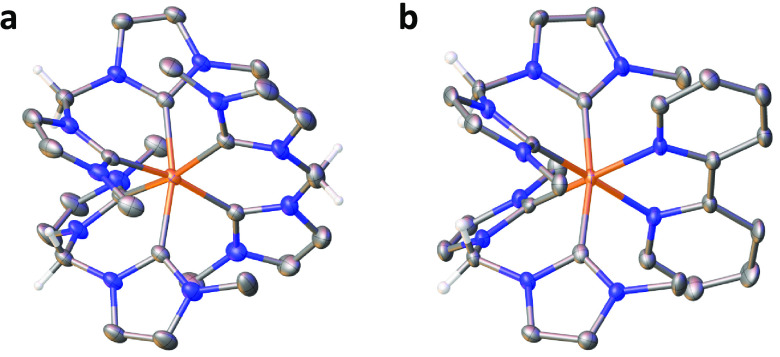
Molecular representations of the X-ray structures of [Fe(mbmi)_3_]^3+^ (a) and [Fe(mbmi)_2_(bpy)]^2+^ (b). Ellipsoids are at 50% and 30%, respectively, with Fe in orange, C in gray, and N in blue. Hydrogen atoms, counteranions, and cocrystallizing solvents are omitted for clarity (hydrogen atoms on the methylene bridges are shown).

Crystals suitable for scXRD were grown by the slow diffusion of diethyl ether into an acetonitrile solution at room temperature. The crystal structures of [Fe(mbmi)_3_](PF_6_)_3_ and [Fe(mbmi)_2_(bpy)](PF_6_)_2_ are shown in parts a and b of Figure 2, respectively.

The structure of [Fe^II^(mbmi)_2_(bpy)](PF_6_)_2_ resembles that of [Fe^II^(btz)_2_(bpy)](PF_6_)_2_^30^ in most aspects. Apart from less planar ligands and wider bite angles for the mbmi ligands compared to btz, other geometric values are comparable. They exhibit the same Fe–L bond lengths (1.96–2.02 vs 1.95–2.01 Å), indicating similar donation of electron density from the NHC ligands to the iron center. [Fe^III^(mbmi)_3_](PF_6_)_3_ does not compare as well with [Fe^III^(btz)_3_](PF_6_)_3_,^40^ where the Fe–L bond lengths go from 1.94–1.98 Å for [Fe^III^(btz)_3_](PF_6_)_3_ to 2.01–2.06 Å for [Fe^III^(mbmi)_3_](PF_6_)_3_. Because the Fe–C bond lengths of [Fe^III^(btz)_3_](PF_6_)_3_ compare well to those of both of the iron(II) complexes and the effect of the formal charge at the iron center seems negligible, the observed Fe–L bond elongation when it comes to [Fe^III^(mbmi)_3_](PF_6_)_3_ is best explained through steric arguments, where three bidentate ligands with an sp^3^-hybridized bridging carbon atom are forced further away from the metal.

[Fe^III^(btz)_3_](PF_6_)_3_^40^ and [Fe^II^(btz)_2_(bpy)](PF_6_)_2_^30^ form five-membered chelate rings between the ligands and metal, while the mbmi ligands of both [Fe^III^(mbmi)_3_](PF_6_)_3_ and [Fe^II^(mbmi)_2_(bpy)](PF_6_)_2_ form six-membered chelate rings upon coordination with the metal center, via the two carbene moieties, after the introduction of the sp^3^-hybridized bridging carbon atom. This difference is reflected in the increased intraligand C–Fe–C angle (bite angle) from ∼79° for [Fe^III^(btz)_3_](PF_6_)_3_ and [Fe^II^(btz)_2_(bpy)](PF_6_)_2_ to ∼85° for [Fe^III^(mbmi)_3_](PF_6_)_3_ (∼89° for one ligand) and [Fe^II^(mbmi)_2_(bpy)](PF_6_)_2_, closer to the ideal octahedral angle of 90° (Table 1).

**Table 1:** Selected Structural Bond Lengths (Fe–L) and Bond Angles (L–Fe–L) of [Fe^III^(mbmi)_3_](PF_6_)_3_ and [Fe^II^(mbmi)_2_(bpy)](PF_6_)_2_ Compared to the Previously Reported [Fe^III^(btz)_3_](PF_6_)_3_^40^ and [Fe^II^(btz)_2_(bpy)](PF_6_)_2_,^30^ Respectively^a^

	[Fe^III^(btz)_3_](PF_6_)_3_^40^	[Fe^III^(mbmi)_3_](PF_6_)_3_	[Fe^II^(btz)_2_(bpy)](PF_6_)_2_^30^	[Fe^II^(mbmi)_2_(bpy)](PF_6_)_2_
Fe–C (Å)	1.94–1.98	2.01–2.06	1.96–2.02	1.95–2.01
Fe–N (Å)			1.99–2.00	2.000
C–Fe–C(cis) (deg) intraligand (bite angle)	79.2	85.4–89.5	79.3–80.0	85.5–85.9
N–Fe–N (deg)			80.45	79.57
C–Fe–C(trans) (deg)	179.0	166.2–166.8	172.6	169.7
C–Fe–N(trans) (deg)			172.7–178.1	170.6–172.0

a All individual values can be found in Table S1.

The geometries of [Fe(mbmi)_3_](PF_6_)_3_ and [Fe(mbmi)_2_(bpy)](PF_6_)_2_ were further analyzed using octahedricity (*O*) and planarity (*P*) factors, as applied by Lundquist^66^ and later by Österman et al.^67^ and Fredin et al.^68^ (Table 2), calculated as the root-mean-square error (RMSE) from ideal L–Fe–L bond angles and dihedral angles between the planes of the two heterocycles cycles of each ligand, respectively. If the ligand sphere is octahedral, all L–Fe–L angles are 90° (*O*_cis_) or 180° (*O*_trans_) for cis- and trans-positioned ligands, respectively, and *O* = 0. Similarly, flat ligands would exhibit a dihedral angle of 0° and *P* = 0. By accommodating extended bite angles, the mbmi ligands reduce the deviation from 90° cis angles in their respective complexes, resulting in lower *O*_cis_ values than the btz complexes (entry 1 in Table 2). However, the deviation from 180° trans L–Fe–L angles (*O*_trans_) is significantly larger for both mbmi complexes, compared to their btz counterparts (entry 2 in Table 2), as a result of the increased ligand flexibility. The overall octahedricity (*O*_total_, entry 3 in Table 2), when all 15 L–Fe–L angles are given the same weight, is less (higher *O*) for [Fe(mbmi)_3_](PF_6_)_3_ than for [Fe(btz)_3_](PF_6_)_3_, while the *O*_total_ values for [Fe(mbmi)_2_(bpy)](PF_6_)_2_ and [Fe(btz)_2_(bpy)](PF_6_)_2_ are comparable. Although the bridging, sp^3^-hybridized central carbon atom of the mbmi ligand allows for extended bite angles, the increased structural flexibility also introduces the possibility of ligand twist, which is interestingly only seen for two out of three ligands (dihedral angles: 50.23 and 50.27°) in the homoleptic [Fe(mbmi)_3_](PF_6_)_3_ complex, while the third mbmi ligand is close to planar between the two imidazole planes (dihedral angle: 4.61°) (Figure S6). This results in a drastic increase of *P* (less planar ligands) compared to [Fe(btz)_3_](PF_6_)_3_, with conjugated, planar btz ligands. Roughly the same geometry is found for the heteroleptic complex [Fe(mbmi)_2_(bpy)](PF_6_)_2_, where coordination slightly twists the bpy ligand from its planarity (dihedral angle: 7.28°), while the two NHC ligands are more significantly twisted (dihedral angles: 46.44 and 49.99°) (Figure S7) and contribute most to the relatively high *P* value of [Fe(mbmi)_2_(bpy)](PF_6_)_2_.

**Table 2:** Geometrical Values for [Fe^III^(mbmi)_3_](PF_6_)_3_, [Fe^II^(mbmi)_2_(bpy)](PF_6_)_2_, [Fe^III^(btz)_3_](PF_6_)_3_ and [Fe^II^(btz)_2_(bpy)](PF_6_)_2_, Calculated through the RMSE from Ideal L–Fe–L Bond Angles (*O*_cis_, 90°; *O*_trans_, 180°) and Dihedral Angles between the Two Heterocycles of Each Ideally Planar Ligand, Respectively^66−68^

	[Fe^III^(btz)_3_](PF_6_)_3_^40^	[Fe^III^(mbmi)_3_](PF_6_)_3_	[Fe^II^(btz)_2_(bpy)] (PF_6_)_2_^30^	[Fe^II^(mbmi)_2_ (bpy)](PF_6_)_2_
*O*_cis_	9.61	8.32	7.13	6.11
*O*_trans_	0.95	13.4	6.09	9.28
*O*_total_	8.61	9.56	6.93	6.86
*P*	3.23	41.1	5.80	39.6

### Steady-State Absorption Spectroscopy

**Figure 3:**
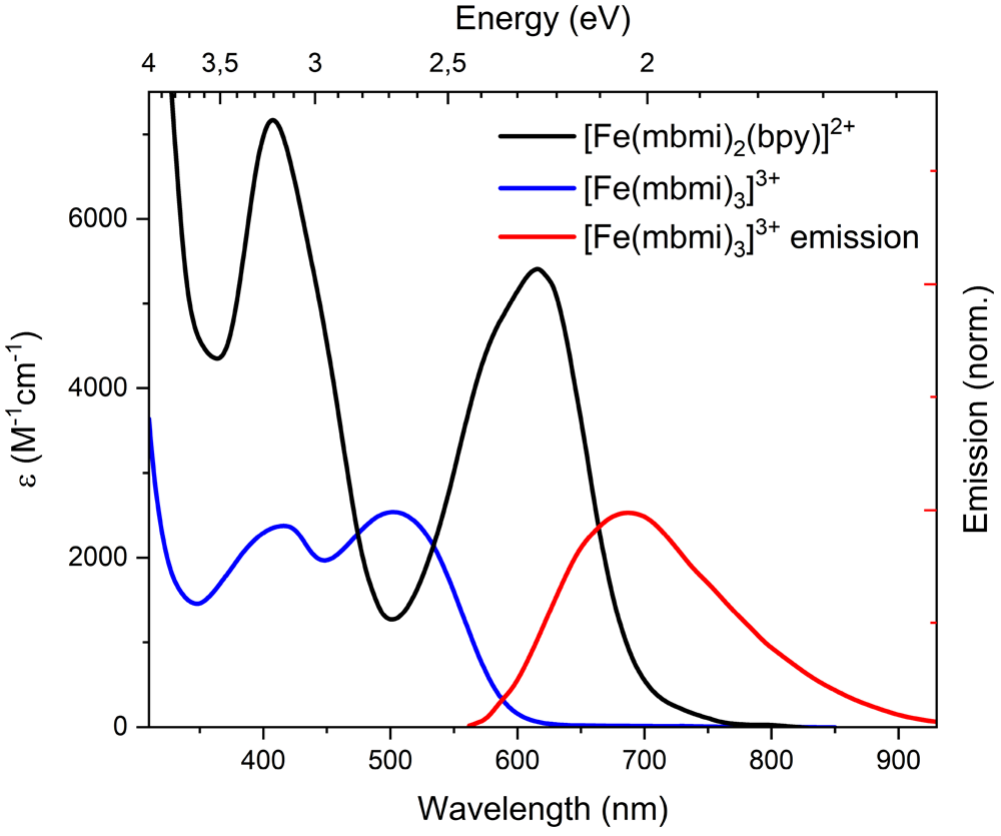
Linear absorption spectra of [Fe(mbmi)_2_(bpy)]^2+^ (black, 480 μM) and [Fe(mbmi)_3_]^3+^ (blue, 560 μM) in acetonitrile together with the emission spectrum of [Fe(mbmi)_3_]^3+^ recorded with 500 nm excitation in dry and deaerated acetonitrile (red, scaled).

Linear absorption was measured in dry acetonitrile at concentrations of 560 μM ([Fe(mbmi)_3_]^3+^) and 480 μM ([Fe(mbmi)_2_(bpy)]^2+^) in a 1 mm cuvette (Figure 3).

For [Fe(mbmi)_3_]^3+^, the linear absorption (blue in Figure 3; the main results are summarized in Table 3) exhibits two pronounced features in the visible region, at 420 nm (2.95 eV) and 502 nm (2.47 eV), while remaining featureless for wavelengths longer than 700 nm. For wavelengths shorter than 350 nm, the absorption rises rapidly, peaking at 254 nm (4.88 eV). These results can be analyzed based on the assignments done for [Fe(btz)_3_]^3+^.^40^ The peak at 254 nm can be attributed to ligand π–π* transitions. The lowest-energy absorption band peaking at 502 nm is assigned to a LMCT transition, and the absorption band at 420 nm could correspond to a second LMCT band. These assignments are consistent with the magnitude of the molar extinction coefficients and are supported by spectroelectrochemistry data (see below). The blue shift of 56 nm in the LMCT transition in going from [Fe(btz)_3_]^3+^ to [Fe(mbmi)_3_]^3+^ could be explained by the lowering of the highest occupied ligand molecular orbital energy due to the breaking of the π conjugation in the mbmi ligand. In the case of [Fe(mbmi)_2_(bpy)]^2+^ (black in Figure 3; the main results are summarized in Table 3), three peaks are found at 616 nm (2.01 eV), 410 nm (3.02 eV), and 304 nm (4.08 eV), with the two low-energy features being quite broad. The higher-energy feature (304 nm) is composed of two peaks separated by ∼0.1 eV. Based on the assignments done for [Fe(btz)_2_(bpy)]^2+^,^30^ the absorption spectrum of [Fe(mbmi)_2_(bpy)]^2+^ can be tentatively assigned. Hence, the band peaking at 304 nm is assigned to π–π* transitions, and the lowest-energy band peaking at 619 nm is assigned to a MLCT transition and involves the bpy ligand, the latter according to the spectroelectrochemistry data and quantum-chemical calculations (see below).

**Table 3:** Comparison of the Photophysical Properties of Complexes Involved in This Study^a^

	[Fe^III^(btz)_3_]^3+^^40^	[Fe^III^(mbmi)_3_]^3+^	[Fe^II^(btz)_2_(bpy)]^2+^^30^	[Fe^II^(mbmi)_2_(bpy)]^2+^
excited state	^2^LMCT	^2^LMCT	^3^MLCT	^3^MLCT
absorption λ_max_ (nm)	384, 528, 558	254, 420, 502	300, 432, 609	304, 410, 616
extinction coefficient ε (M^–1^ cm^–1^)	1500 (528 nm)	2540 (502 nm)	3260 (609 nm)	5410 (616 nm)
excited-state lifetime τ (ps)	100	57	13	7.6
photoluminescence λ_em_ (nm)	600	686	NA	NA
quantum yield Φ_e_ (in CH_3_CN)	(3 ± 0.5) × 10^–4^	(1.2 ± 0.1) × 10^–4^	NA	NA

a All measurements were carried out in deaerated acetonitrile.

### Steady-State Emission Spectroscopy

**Figure 4:**
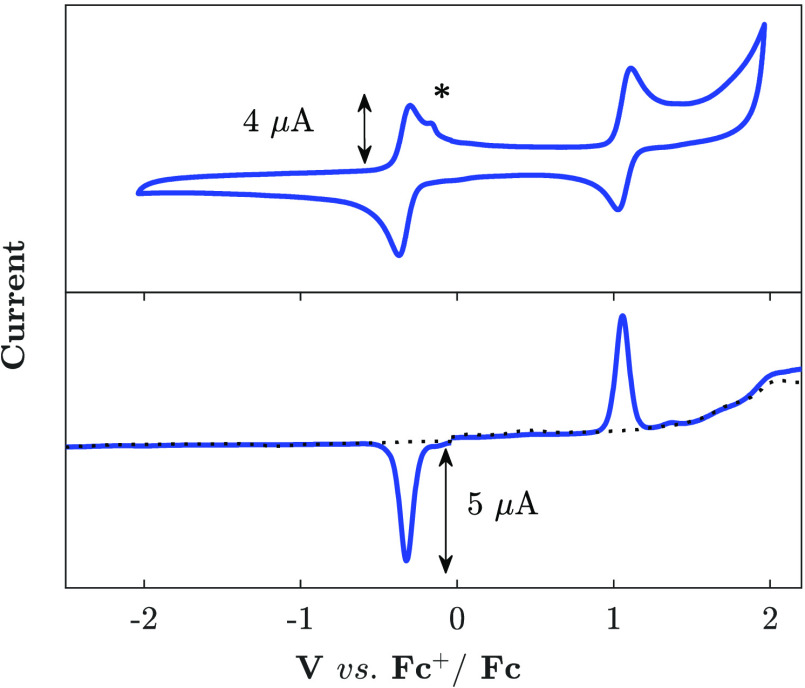
Cyclic (top) and differential pulse voltammograms (bottom) of [Fe(mbmi)_3_]^3+^ (1 mM) in acetonitrile (0.1 M TBAPF_6_). The feature marked with an asterisk is an silver desorption peak caused by Ag^+^ ion leakage from the reference electrode.

[Fe(mbmi)_3_]^3+^ was dissolved in acetonitrile and filtered (0.45 μm poly(tetrafluoroethylene) filter; *C* = 200 μM). After [Fe(mbmi)_3_]^3+^ was excited at 500 nm (2.48 eV; the lowest-energy feature of the absorption; Figure 3, blue), a broad emission centered at 686 nm (1.81 eV; Figure 4, red; the results are listed in Table 3) was found.^40^ The quantum yield (Φ_e_) was determined to be (1.2 ± 0.1) × 10^–4^ (for details, see Supporting Information section S10), which is somewhat lower than the quantum yield of (3 ± 0.5) × 10^–4^ for [Fe(btz)_3_]^3+^.^40^ The peak-to-peak difference from absorption and emission (Stokes shift) corresponds to an energy difference of 0.66 eV (184 nm) (Figure S10), compared to 0.166 eV (42 nm) for [Fe(btz)__3__]^3+^. Taken together, the emission quantum yield (Φ_e_) and excited-state lifetime (τ) provide a radiative rate constant of *k*_r_ = Φ_e_/τ = 2.1 × 10^6^ s^–1^. Integration of the LMCT (502 nm) band between 455 and 605 nm results in an integrated extinction coefficient of *A* ≈ 10^7^ M^–1^ cm^–2^ from which a radiative rate constant of *k*_r_ ≈ 2 × 10^7^ s^–1^ is predicted with the Strickler–Berg relationship. Considering the significant overlap of the 502 nm absorption band with the higher-energy absorption band, the agreement between the observed value of *k*_r_ and the estimate based on the intensity of the absoprtion band seems reasonable. This result suggests that the emission occurs directly from the ^2^LMCT state, with *E*_0–0_ = 2.1 eV provided by the intersection of normalized absorption and emission bands at 588 nm (Figure 3).

The reduced quantum yield of [Fe(mbmi)_3_]^3+^ compared to [Fe(btz)_3_]^3+^ can be related to a somewhat more efficient nonradiative decay in the former complex. This is, in turn, also consistent with a more significant excited-state relaxation of [Fe(mbmi)_3_]^3+^ compared to [Fe(btz)_3_]^3+^, which manifests itself in the larger observed Stokes shift mentioned above and a longer emission wavelength of 696 nm for [Fe(mbmi)_3_]^3+^ compared to 600 nm for [Fe(btz)_3_]^3+^. To confirm that the emission is correlated to the LMCT absorption, luminescence excitation measurement was performed (Figure S10). No emission was detected for [Fe(mbmi)_2_(bpy)]^2+^, which parallels the findings for [Fe(btz)_2_(bpy)](PF_6_)_2_.^30^

### Cyclic Voltammetry and Spectroelectrochemistry

The voltammograms of [Fe(mbmi)_3_]^3+^ in acetonitrile (Figure 4) show reversible one-electron waves at *E*_1/2_ = −0.38 and 1.03 V (all potentials vs Fc^+/0^), which can be assigned to the Fe^III/II^ and Fe^IV/III^ couples, respectively. Within the electrolyte window, oxidation or reduction of the mbmi ligand can only be observed as the onset of additional waves above +2 V and below −3 V. Efficient stabilization of the Fe^IV^ state by the mbmi ligand (cf. the 1.16 V irreversible peak potential for [Fe(btz)_3_]^3+^)^38^ in combination with the difficult oxidation of the mbmi ligand results in a reversible Fe^IV/III^ wave well resolved from ligand oxidation and electrolyte breakdown previously only observed with [Fe^III^(phtmeimb)_2_]^+^ and its derivatives that feature Fe^IV/III^ potentials below 0.3 V.^41,65^ This enabled the clean electrochemical in situ formation of [Fe(mbmi)_3_]^4+^ and the spectroelectrochemical characterization of all three metal oxidation states.

**Figure 5:**
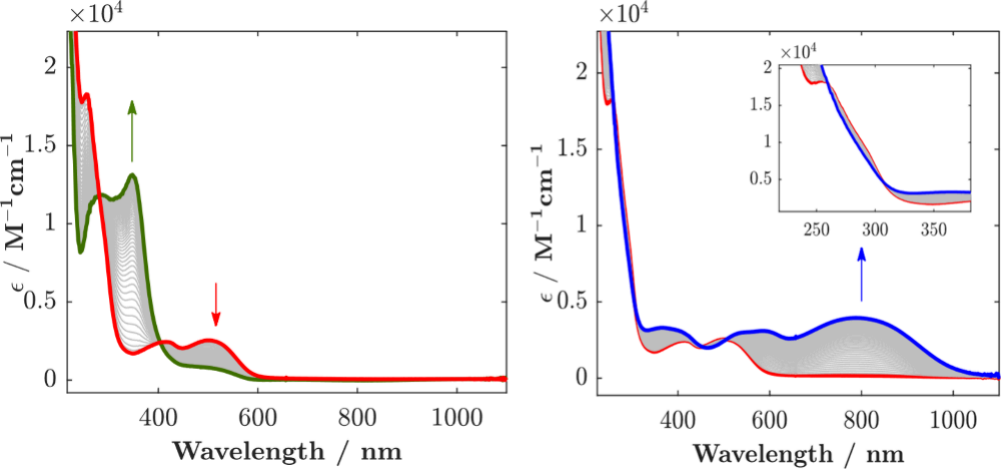
Spectroelectrochemistry of [Fe(mbmi)_3_]^3+^ in acetonitrile: (left) reduction at −0.8 V; (right) oxidation at 1.4 V. [Fe(mbmi)_3_]^2+^ (green —), [Fe(mbmi)_3_]^3+^ (red —), and [Fe(mbmi)_3_]^4+^ (blue —). Inset: Spectral changes in the UV.

The position of the lowest-energy absorption band of [Fe(mbmi)_3_]^3+^ (Figure 5) peaking at 502 nm (ε = 2540 M^–1^ cm^–1^) agrees reasonably well with the potential difference between the Fe^III/II^ couple and ligand oxidation, suggesting a LMCT transition. Controlled potential reduction of [Fe(mbmi)_3_]^3+^ at −0.80 V results in reversible bleaching of this LMCT band with clear isosbestic points at 404 and 282 nm, supporting the assignment of the redox process to the metal center. The formed Fe^II^ state has a high-energy absorption band, peaking at 348 nm (ε = 13140 M^–1^ cm^–1^), which can be assigned to a MLCT transition consistent with the potentials of the Fe^III/II^ couple and ligand reduction below −3 V. On the other hand, oxidation of [Fe(mbmi)_3_]^3+^ at 1.40 V yields a panchromatic absorption across the visible region. The spectral changes are reversible, with isosbestic points at 260, 307, 453, and 506 nm, and are attributed to the formation of [Fe(mbmi)_3_]^4+^. The assignment is corroborated by the position of the lowest-energy absorption band centered at 789 nm (ε = 3950 M^–1^ cm^–1^), which can be expected for a LMCT transition, based on the potentials of the Fe^IV/III^ couple and ligand oxidation.

**Figure 6:**
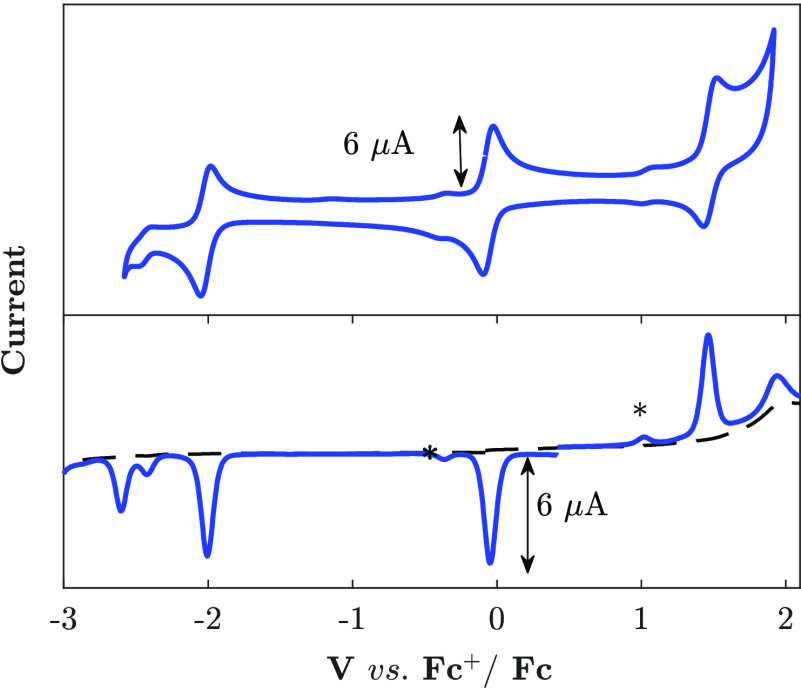
Cyclic (top) and differential pulse voltammograms (bottom) of [Fe(mbmi)_2_(bpy)]^2+^ (0.98 mM) in acetonitrile (0.1 M TBAPF_6_; the features marked with asterisks are due to a minor [Fe(mbmi)_3_]^3+^ contamination).

The voltammograms of [Fe(mbmi)_2_(bpy)]^2+^ (Figure 6) show two reversible one-electron oxidation waves, which can be attributed to the Fe^III/II^ couple (*E*_1/2_ = −0.057 V) and the Fe ^IV/III^ couple (*E*_1/2_ = 1.48 V) and reveal destabilization of the higher oxidation states relative to [Fe(mbmi)_3_]^3+^ by about 0.5 eV due to the less electron-donating bpy ligand. At more extreme potentials, irreversible oxidation [1.94 V differential pulse voltammetry (DPV) peak potential] and reversible reduction (*E*_1/2_ = −2.02 V) waves, followed by irreversible reductions (−2.42 and −2.60 V DPV peak potential), are observed, consistent with the potentials of NHC ligand oxidation and bpy reduction in previously studied complexes with similar mixed NHC/bpy ligand motifs.^30^ The reversible first reduction of [Fe(mbmi)_2_(bpy)]^2+^ can be safely attributed to the reduction of the bpy ligand (Figure 6). Its potential (−2.02 V) is significantly less negative than the first, most likely bpy-based reduction of [Fe(btz)_2_(bpy)]^2+^ (−2.28 V) that is only poorly resolved from the subsequent, presumably btz-based reduction.^30^ An even more negative potential of the bpy/bpy^•–^ couple was found for [Fe(CN)_4_(bpy)]^2–^, where the strongly electron-donating CN^–^ ligands place the reversible bpy reduction wave at −2.47 V.^30^ The increasing electron-donating effect of the ligands [(mbmi)_2_) < (btz)_2_ < (CN)_4_] as reflected the bpy/bpy^•–^ reduction potentials give rise to a parallel trend for the Fe^III/II^ couple (−0.06, −0.35, and −0.63 V).

**Figure 7:**
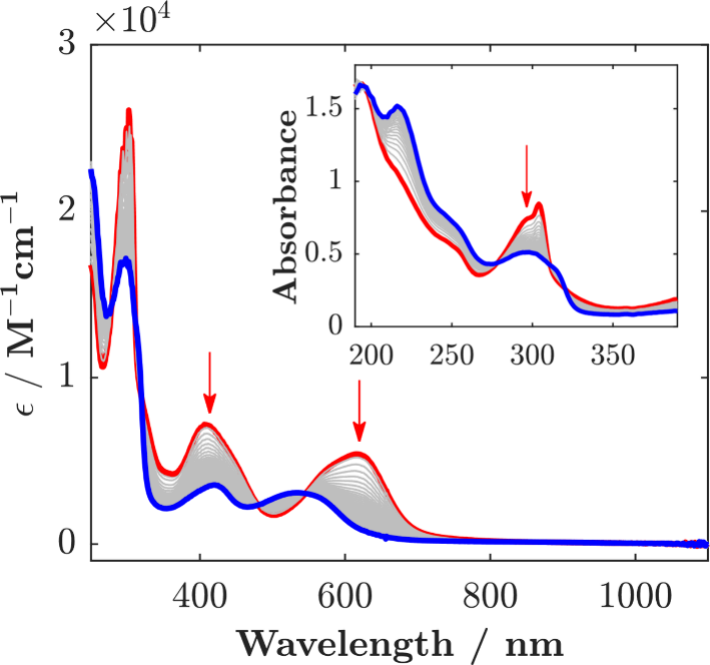
Spectroelectrochemistry of [Fe(mbmi)_2_(bpy)]^2+^ in acetonitrile: oxidation at 0.4 V. [Fe(mbmi)_2_(bpy)]^2+^ (red —) and [Fe(mbmi)_2_(bpy)]^3+^ (blue —). Inset: Spectral changes in the UV at a diluted concentration (0.3 mM in acetonitrile).

The 616 nm peak (ε = 5410 M^–1^ cm^–1^) observed in the absorption spectrum of [Fe(mbmi)_2_(bpy)]^2+^ (Figure 7) can be attributed to a MLCT transition, consistent with the measured electrochemical potential difference between the Fe^III/II^ couple and the first ligand reduction. Oxidation at 0.40 V results in reversible spectral changes with clear isosbestic points at 278, 311, 320, 480, and 545 nm. The lowest-energy absorption band of the resulting Fe^III^ state at 545 nm (ε = 3050 M^–1^ cm^–1^) can be attributed to a LMCT transition based on reasonable agreement with the energy expected from the potential difference between the Fe^III/II^ couple and ligand oxidation.

**Figure 8:**
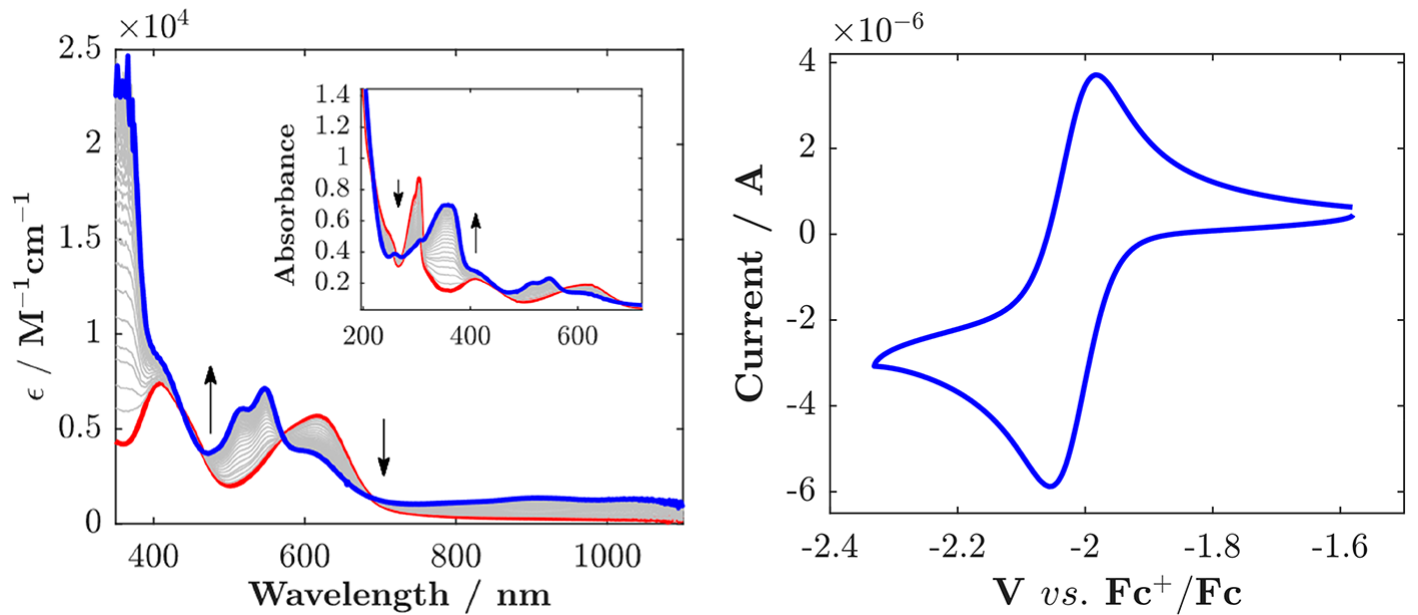
Left: Spectroelectrochemistry of [Fe(mbmi)_2_(bpy)]^2+^; reduction at −2.30 V. [Fe(mbmi)_2_(bpy)]^2+^ (red —), [Fe(mbmi)_2_(bpy)]^+^ (blue —). Inset: Spectral changes in the blue at a diluted concentration. Right: Reversible ligand reduction wave assigned to the bpy/bpy^•−^ couple.

While robust spectroelectrochemical characterization of the Fe^IV/III^ couple of [Fe(mbmi)_2_(bpy)]^2+^ is precluded by the underlying onset of solvent/electrolyte breakdown, the product of the reversible ligand-based reduction could be characterized by spectroelectrochemistry (Figure 8). Reduction at −2.30 V resulted in reversible spectral changes with sharp isosbestic points (219, 259, 275, 311, 450, 572, and 680 nm) that can be assigned to the formation of a stable ligand reduced product. Its pronounced absorption bands at 350–370 nm and the broad NIR absorption together with bleaching of the MLCT band are consistent with a bpy-based reduction. The absorption changes induced by the MC oxidation of Fe(mbmi)_2_(bpy)]^2+^ and reduction of its bpy ligand are shown in Figure 8 as proxy for the expected absorption changes upon excitation to the lowest MLCT excited state.

**Figure 9:**
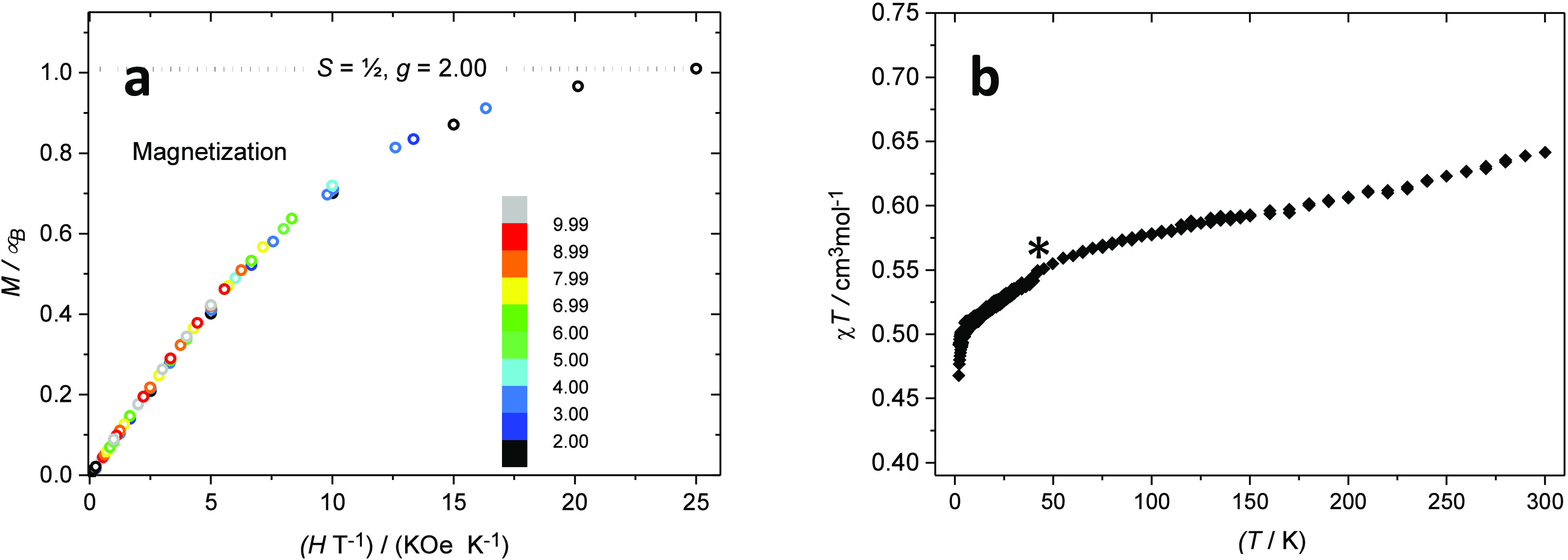
Magnetization data of [Fe(mbmi)_3_]^3+^ (a) recorded at fields of 0.1–5 T and temperatures of 2–10 K. The inset color coding identifies the temperature. The superimposable curves for all fields is expected for a nonzero-field-split *S* = ^1^/_2_ spin system. (b) Magnetic susceptibility versus temperature indicative of an *S* = ^1^/_2_ system with incomplete quenching of the orbital moment. *The bump around *T* = 50 K is due to a small amount of adsorbed dioxygen.

The electrochemical data for [Fe(mbmi)_2_(bpy)]^2+^ and [Fe(mbmi)_3_]^3+^ are summarized in Table 4. Compared to [Fe(btz)_2_(bpy)]^2+^^30^ and [Fe(btz)_3_]^3+^,^38^ the potentials of the Fe^III/II^ couples indicate significantly weaker electron donation by the mbmi ligand. Based on the excited-state energy (*E*_0–0_ = 2.08 eV; see above) of [Fe(mbmi)_3_]^3+^, its excited-state potentials for oxidative [*E*_1/2_(IV/*III) = −1.05 V] and reductive quenching [*E*_1/2_(*III/II) = 1.70 V] of [Fe(mbmi)_3_]^3+^ are similar to those of [Fe(btz)_3_]^3+^ [*E*_0–0_ = 2.18 eV, *E*_1/2_(IV/*III) = −1.0 V, and *E*_1/2_(*III/II) = 1.5 V].^32,33,37,40^ With a lifetime of 57 ps of its ^2^LMCT state (see below), bimolecular excited-state reactions of [Fe(mbmi)_3_]^3+^ would, however, be more difficult to implement than those of [Fe(btz)_3_]^3+^ (100 ps).^40^

**Table 4:** Reduction Potentials of [Fe^III^(btz)_3_](PF_6_)_3_,^40^ [Fe^III^(mbmi)_3_](PF_6_)_3_, [Fe^II^(btz)_2_(bpy)](PF_6_)_2_,^30^ and [Fe^II^(mbmi)_2_(bpy)](PF_6_)_2_ in Acetonitrile

	*E*_1/2_/V vs Fc^+/0^	
	[Fe^III^(btz)_3_](PF_6_)_3_	[Fe^III^(mbmi)_3_](PF_6_)_3_
Fe^III/II^	–0.58	–0.38
Fe^IV/III^	1.16^a^	1.03

	*E*_1/2_/V vs Fc^+/0^	
	[Fe^II^(btz)_2_(bpy)](PF_6_)_2_	[Fe^II^(mbmi)_2_(bpy)](PF_6_)_2_
Fe^II^ L/L^•–^	–2.28^a^	–2.02
Fe^III/II^	–0.35	–0.06
Fe^IV/III^	1.36^a^	1.48

a Irreversible, DPV peak potential.

### Magnetization

The magnetic susceptibility and magnetization for [Fe(mbmi)_3_]^3+^ is reported in Figure 9 and Table 5. The magnetic properties are similar to those of the previously reported [Fe^III^(btz)_3_](PF_6_)_3_.^40^ The magnetization of [Fe^III^(mbmi)_3_](PF_6_)_3_ has been determined in a wider temperature range, yet the lack of nesting of the magnetization curves indicates a system without significant zero-field splitting. The temperature variation of the magnetic susceptibility suggests a largely, but incompletely quenched orbital momentum. The formulation of the complex as a low-spin Fe^III^ is thus corroborated by these magnetic data.

**Table 5:** Comparison of the Experimental Values from Magnetization and Mössbauer Measurements (at 80 K) of [Fe^III^(mbmi)_3_](PF_6_)_3_ and [Fe^II^(mbmi)_2_(bpy)](PF_6_)_2_

	[Fe^III^(btz)_3_](PF_6_)_3_^40^	[Fe^III^(mbmi)_3_](PF_6_)_3_	[Fe^II^(btz)_2_(bpy)](PF_6_)_2_^30^	[Fe^II^(mbmi)_2_(bpy)](PF_6_)_2_
magnetization	*S* = ^1^/_2_, *g*_av_ ≈ 2.2	*S* = ^1^/_2_, *g*_av_ ≈ 2.0	NA	NA
Mössbauer (CS, QS) (mm s^–1^)	(0.03, 2.22)	(0.05, 1.33)	(0.16, 0.83)	(0.18, 0.63)

### Mössbauer Study

**Figure 10:**
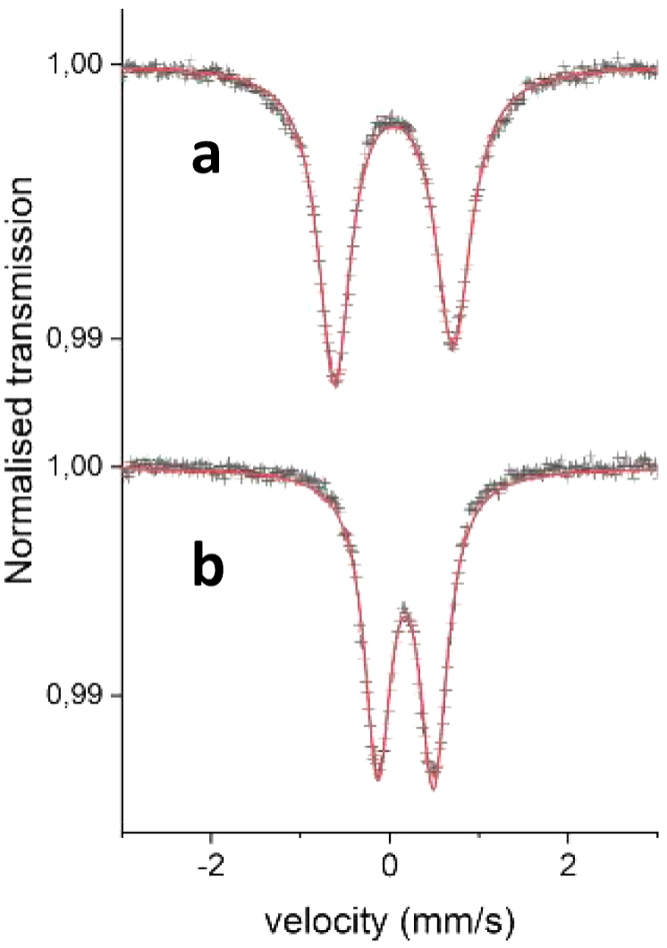
^57^Fe Mössbauer spectra of (a) [Fe(mbmi)_3_]^3+^ and (b) [Fe(mbmi)_2_(bpy)]^2+^, recorded at 80 K.

**Figure 11:**
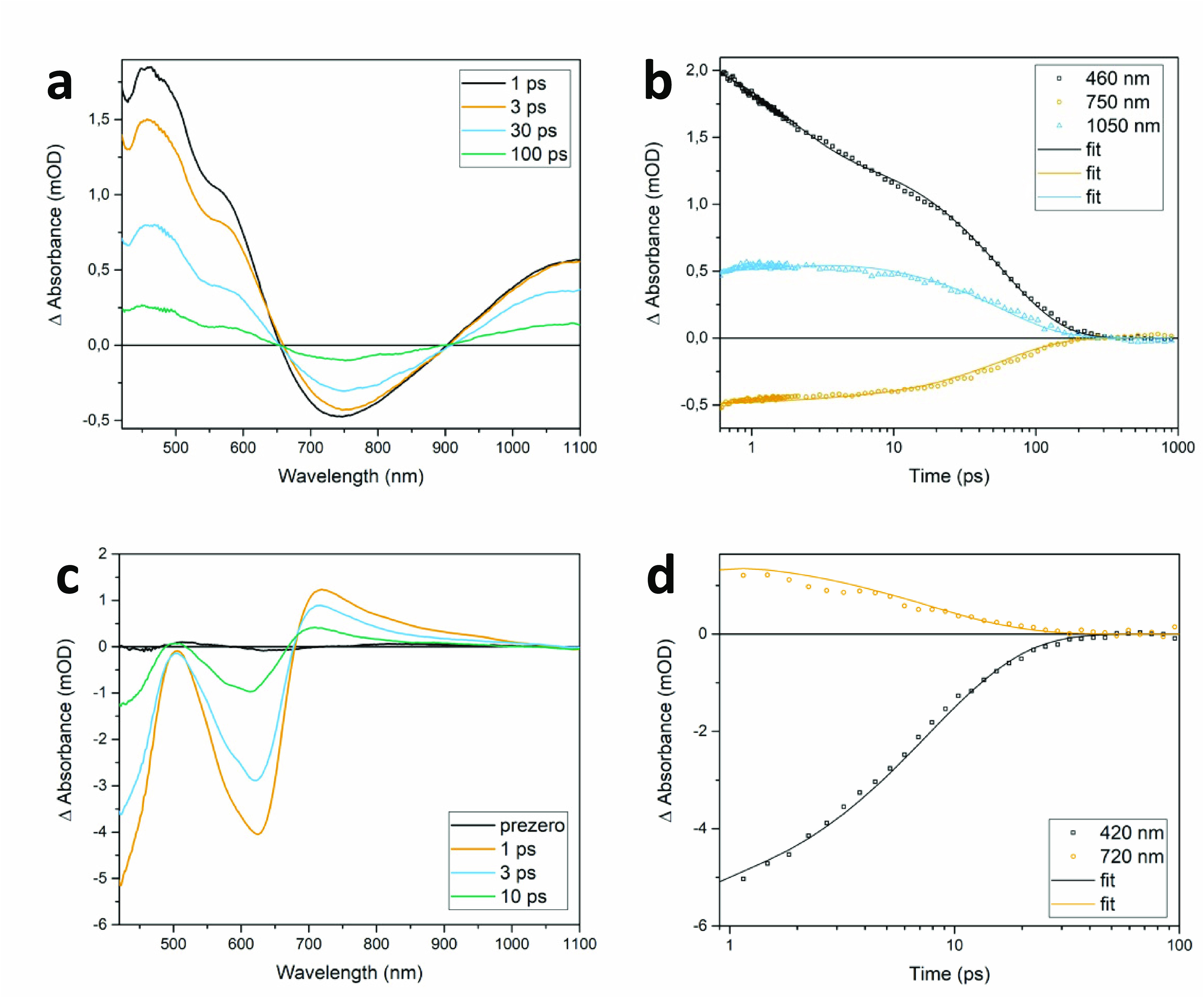
(a) TA spectra of [Fe(mbmi)_3_]^3+^. Excitation at 400 nm. (b) Kinetics of [Fe(mbmi)_3_]^3+^. (c) TA spectra of [Fe(mbmi)_2_(bpy)]^2+^. Excitation at 620 nm. (d) Kinetics of [Fe(mbmi)_2_(bpy)]^2+^. All measurements were carried out in dry and deaerated acetonitrile. The open symbols represent the experimental data; the solid lines are fits.

Mössbauer spectra of [Fe(mbmi)_3_]^3+^ and [Fe(mbmi)_2_(bpy)]^2+^ at 80 and 295 K show resolved doublet structures (Figure 10). The isomer chemical shift (CS) and electric quadrupole splitting (QS) of the doublets are [0.047(5), 1.330(5)] and [0.179(5), 0.627(5)] mm s^–1^ at 80 K, respectively. Their Lorentzian full width at half-maximum (fwhm) values are 0.483(10) and 0.372(10) mm s^–1^ at 80 K, respectively (Table 5). The doublet for [Fe(mbmi)_3_]^3+^ reveals broad lines with some line asymmetry. At room temperature, the parameters found for (CS, QS) are (−0.04, 1.12) and (0.12, 0.58) mm s^–1^, respectively. A much lower Mössbauer recoil free fraction was observed for [Fe(mbmi)_2_(bpy)]^2+^ compared to [Fe(mbmi)_3_]^3+^ at 295 K. The errors for all hyperfine parameters are 0.01 mm s^–1^. The combination of the CS and QS values shows quite clearly that Fe in [Fe(mbmi)_3_]^3+^ is in a low-spin Fe^III^ state, whereas Fe in [Fe(mbmi)_2_(bpy)]^2+^ is in a low-spin Fe^II^ state (Figure 11). The asymmetry of the Fe^III^ doublet found at 80 K can be explained on the basis of magnetic relaxation effects.^69^ The strong temperature dependence of the Fe^II^ Mössbauer signal compared to the Fe^III^ signal furthermore reveals a difference in the Debye temperatures θ_D_ for the two Fe valences in these complexes in line with earlier findings.^32,40,41^

### Electron Paramagnetic Resonance (EPR) Spectroscopy

X-band EPR spectroscopy was applied to further investigate the spin properties of [Fe^II^(mbmi)_2_(bpy)](PF_6_)_2_ and [Fe^III^(mbmi)_3_](PF_6_)_3_. According to our expectations, the low-spin [Fe^II^(mbmi)_2_(bpy)](PF_6_)_2_ is EPR-silent. In contrast, the low-spin [Fe^III^(mbmi)_3_](PF_6_)_3_ has an active spin *S* = ^1^/_2_, as indicated by the SQUID measurement, and is therefore expected to show an EPR spectrum with signals near the *g* – 2 region from a spin transition between the |±^1^/_2_⟩ states. Contrary to our anticipation, [Fe^III^(mbmi)_3_](PF_6_)_3_ did not show an active EPR spectrum (for details and spectra, see Supporting Information section S7). A similar phenomenon was observed and reported in our earlier investigations with Fe^III^ caged in a strong electron-donating NHC ligand.^41,65^ Our observation is in line with theory: For low-spin d^5^ systems, the calculated *g* values are extremely dependent on small changes in the low-symmetry ligand-field components (and vibronic coupling). This phenomenon has been analyzed by Tsukerblat et al.^70,71^ For systems with small, but nonzero, low-symmetry ligand-field components, the distribution in the *g* anisotropies can become so large that detection of the EPR signal becomes impossible.

### Transient Absorption (TA) Spectroscopy

TA spectroscopy was employed to investigate the excited-state dynamics of [Fe(mbmi)_3_]^3+^ and [Fe(mbmi)_2_(bpy)]^2+^. All TA measurements were performed in deaerated acetonitrile in a 1 mm quartz cuvette under ambient conditions, moving the sample after each scan. The stability of each sample was confirmed by comparing the absorption spectra before and after TA experiments, and no photodamage was detected. The pump intensity was kept below 10^15^ photons pulse^–1^ cm^–2^ for both complexes.

[Fe(mbmi)_3_]^3+^ was excited at 400 nm, and resulting TA spectra at various times after excitation are plotted in Figure 11a. The excited-state absorption (ESA) is overlaid by a strong stimulated emission (SE) signal, peaking at ∼750 nm. Ground-state bleach (GSB) is seen only as weak dips in the blue part of the ESA at 420 and 502 nm. Kinetics depicting the dynamics of SE and the ESA of [Fe(mbmi)_3_]^3+^ (Figure 11b) show decay profiles similar to those of the two decay components of 1.6 and 57.3 ps obtained by a global fit, with the latter assigned to the ^2^LMCT state, which has approximately half the lifetime of the respective state in [Fe^III^(btz)_3_](PF_6_)_3_.^40^ The longer excited-state lifetimes of [Fe^III^(btz)_3_](PF_6_)_3_ can be attributed to the combined effect of the stronger electron donation of the btz framework compared to mbmi and the more octahedral environment, leading to a stronger ligand field imposed by the ligand in [Fe^III^(btz)_3_](PF_6_)_3_ compared to [Fe(mbmi)_3_]^3+^, as revealed in the electrochemical investigation above.

[Fe(mbmi)_2_(bpy)]^2+^ was excited at 620 nm, with the resulting TA spectra shown in Figure 11c. Two GSB bands are observed at 410 and 616 nm, and there is noticeably weaker ESA in the red from GSB, peaking at around 725 nm. Kinetics depicting the dynamics in the GSB and ESA regions (Figure 11d) show two decay components of 0.22 and 7.6 ps as a result of a global fit, with the latter assigned to the ^3^MLCT state, showing faster ground-state recovery than the ^3^MLCT of [Fe^II^(btz)_2_(bpy)](PF_6_)_2_.^30^ The longer excited-state lifetimes of [Fe^II^(btz)_2_(bpy)](PF_6_)_2_ can be attributed to the effect of stronger electron donation of the btz framework compared to mbmi, as revealed in the electrochemical investigation above.

### Quantum-Chemical Calculations

**Figure 12:**
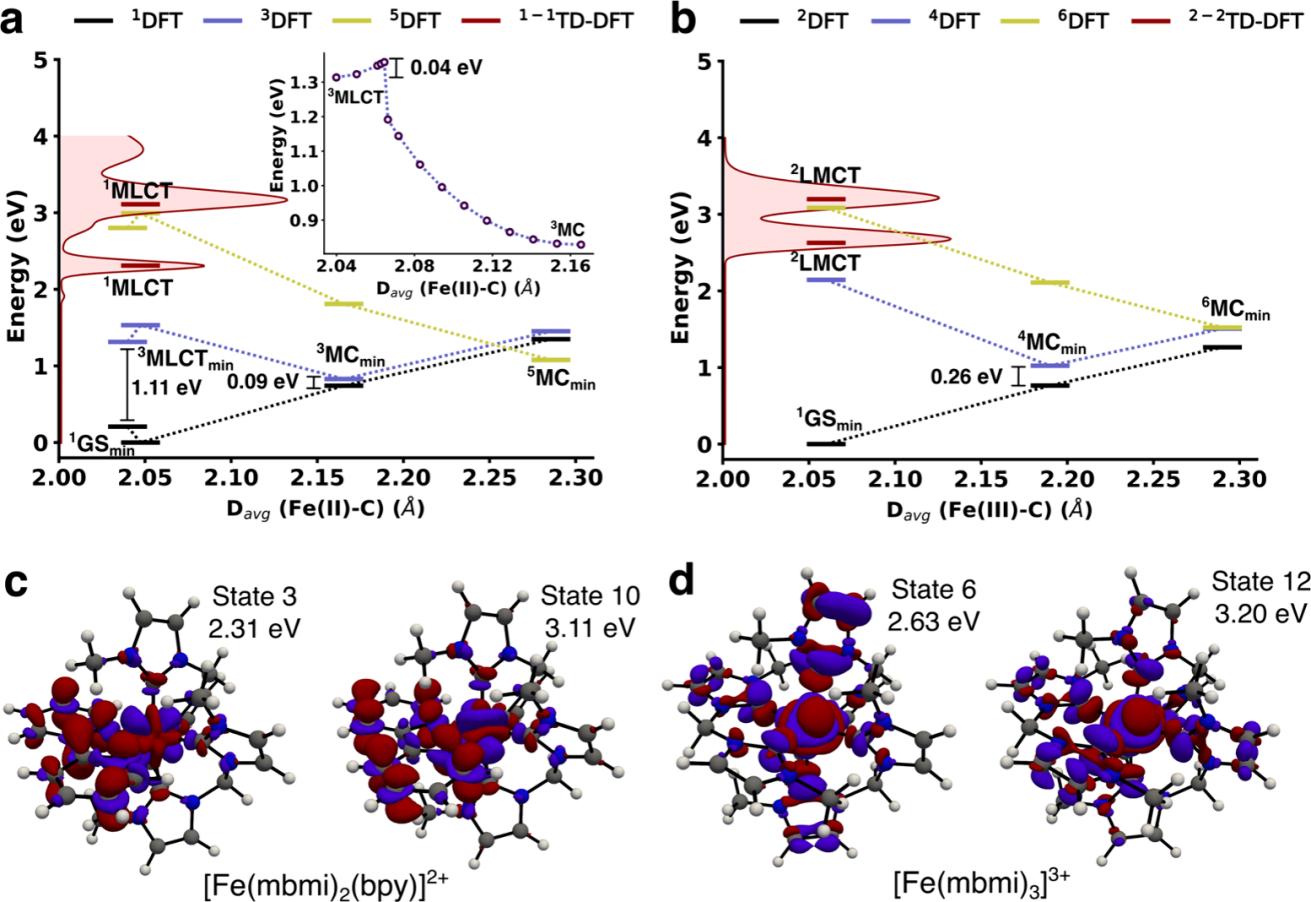
DFT state energy diagrams for (a) [Fe(mbmi)_2_(bpy)]^2+^ showing the lowest singlet, triplet, and quintet states and a minimum-energy-path calculation between the relaxed ^3^MLCT and ^3^MC (inset plot) and (b) [Fe(mbmi)_3_]^3+^ showing the lowest doublet, quartet, and hextet states. TD-DFT vertical excitations, together with the calculated absorption spectra (shown on the left axis), are included. The metal–ligand distances are calculated as the average for the six iron–ligand bonds. All calculations were performed at the B3LYP*/6-311G(d)/PCM (acetonitrile). TD-DFT density difference between the ground state and (c) the singlet excited states 3 and 10 in [Fe(mbmi)_3_]^3+^ and (d) the doublet excited states 6 and 12 in [Fe(mbmi)_2_(bpy)]^2+^. The blue color reflects the density depletion and the red color the density gain.

The calculated electronic state properties of [Fe(mbmi)_2_(bpy)]^2+^ and [Fe(mbmi)_3_]^3+^ are shown in Figure 12a,b, left axis (for data, see Supporting Information section S12), highlighting the influence incurred by the difference in the oxidation state of the central iron in the two complexes. The time-dependent density functional theory (TD-DFT)-calculated UV–vis spectra of [Fe(mbmi)_2_(bpy)]^2+^ and [Fe(mbmi)_3_]^3+^ (Figures S12 and 12a,b) are in good agreement with the reported experimental absorption spectra in Figure 3. The lower-energy band at 2.31 eV and a second intense band at 3.11 eV in the calculated [Fe(mbmi)_2_(bpy)]^2+^ absorption spectrum were identified as ^1^MLCT states. Analysis of the TD-DFT vertical transitions revealed the electron transition origin from the bpy ligand, as displayed in Figure 12c. Two triplet-state minima were identified for [Fe(mbmi)_2_(bpy)]^2+^ with unrestricted DFT and characterized as MC and MLCT states, respectively. The relaxed ^3^MC state, represented in Figure 12c, is calculated to be nearly degenerate with the ground state at the relaxed ^3^MC geometry (energy gap of 0.09 eV). Furthermore, a calculated minimum energy path connecting the CT and MC triplet excited states (Figure 12a, inset) indicates a downhill deactivation process from the CT state with a small activation energy estimated to be ∼0.04 eV. The short-lived excited state in [Fe(mbmi)_2_(bpy)]^2+^ was therefore tentatively attributed to the ^3^MLCT state deactivation via the lower-lying MC state toward the ground state. The quintet MC state minimum structure (^5^MC_min_) was calculated to be more stable than the ground state and ^3^MC at the ^5^MC_min_ geometry. The stronger ligand field in [Fe(mbmi)_3_]^3+^ compared to [Fe(mbmi)_2_(bpy)]^2+^ slightly raises the low-spin ^4^MC vertical energy gap with the doublet ground state to 0.26 eV, as shown in Figure 12b. Due to the computational challenge to reliably characterize the relaxed open-shell ^2^LMCT in [Fe(mbmi)_3_]^3+*n*^, further investigations of this excited state were not attempted here. However, the occurrence of the two intense bands in the calculated absorption spectra at 2.63 and 3.20 eV were identified as two ^2^LMCT excited states (density differences depicted in Figure 12d).

## Conclusion

We report two potential molecular photosensitizers, the homoleptic hexa-NHC [Fe(mbmi)_3_](PF_6_)_3_ and heteroleptic tetra-NHC [Fe(mbmi)_2_(bpy)](PF_6_)_2_ complexes, featuring the nonconjugated mbmi ligand, containing two imidazole NHC moieties, separated by a methylene group. The structural and electronic effects of such ligands on the ground- and excited-state properties of the complexes have been investigated and compared to the previously reported complexes [Fe(btz)_3_](PF_6_)_3_ and [Fe(btz)_2_(bpy)](PF_6_)_2_, respectively, where btz is a conjugated bidentate ligand based on two mesoionic 3-methyl-1-(*p*-tolyl)(1,2,3-triazole-5-ylidene) NHC units. The excited-state dynamics of [Fe(mbmi)_3_](PF_6_)_3_ and [Fe(btz)_3_](PF_6_)_3_ and of [Fe(btz)_2_(bpy)](PF_6_)_2_ and [Fe(mbmi)_2_(bpy)](PF_6_)_2_, respectively, show a direct correlation between the geometry of the two metal complexes in each pair and their CT excited-state properties. The more distorted coordination sphere around the metal center leads to faster nonradiative deactivation of the CT states of [Fe(mbmi)_3_](PF_6_)_3_ and [Fe(mbmi)_2_(bpy)](PF_6_)_2_ compared to the more rigid congeners [Fe(btz)_3_](PF_6_)_3_ and [Fe(btz)_2_(bpy)](PF_6_)_2_, respectively. As a result, the photoluminescence quantum yield was also lower for [Fe(mbmi)_3_](PF_6_)_3_ compared to [Fe(btz)_3_](PF_6_)_3_. The weaker electron donation from the carbene ligand, as is evident from the less reducing (Fe^II^/Fe^III^) redox couples of [Fe(mbmi)_3_](PF_6_)_3_ and [Fe(mbmi)_2_(bpy)](PF_6_)_2_ compared to [Fe(btz)_3_](PF_6_)_3_ and [Fe(btz)_2_(bpy)](PF_6_)_2_, respectively, can be expected to result in less pronounced destabilization of the MC states, which could contribute to the faster nonradiative decay of the CT states.

Importantly, [Fe(mbmi)_3_](PF_6_)_3_, containing the relatively flexible and nonconjugated mbmi ligand, constitutes the fourth structure type reported of an emissive iron(III) complex at room temperature.^40,41,72,73^ In summary, our findings show that higher structural flexibility in the coordination sphere does not necessarily translate to improved geometrical, electronic, or photophysical properties of Fe-NHC complexes, something also observed by Gros et al.^59^ While octahedricity^42,43^ influences their electronic structure and photophysical properties in a predictable fashion, the number and design of NHC ligands does not correlate in a simple fashion with the structure of the resulting Fe-NHC complexes. Our results hopefully encourage continued exploration of this structure–photophysical/electronic relationship in Fe-NHC complexes toward a more complete understanding of the field of iron-based photochemistry.

## Supporting Information Available

Synthesis, ^1^H and ^13^C NMR spectra, HR-MS spectra, scXRD, Mößbauer spectroscopy, magnetization measurements, EPR spectroscopy, steady-state absorption spectroscopy, TA spectroscopy, and quantum chemistry, including figures and tables (PDF)
