# Supplementary material for: How Rigidity and Conjugation of Bidentate Ligands Affect the Geometry and Photophysics of Iron N-Heterocyclic Complexes: A Comparative Study
Source: Inorg Chem. 2024 Feb 29;63(10):4461–73. doi: 10.1021/acs.inorgchem.3c03972 (PMC10934811; doi:10.1021/acs.inorgchem.3c03972)
Supplement: Supplementary file 1 — ic3c03972_si_001.pdf [file ic3c03972_si_001.pdf]

SUPPORTING INFORMATION

How Rigidity and Conjugation of Bidentate Ligands Affect the Geometry and Photophysics of Iron *N*-heterocyclic Complexes – A Comparative Study

Om Prakash,[+a] Pavel Chábera,[+b] Nidhi Kaul,[+c] Valtýr F. Hlynsson,[+a] Nils W. Rosemann,[b] Iria Bolaño Losada,[d] Yen Tran Hoang Hai,[d] Ping Huang,[c] Jesper Bendix,[e] Tore Ericsson,[e] Lennart Häggström,[f] Arvind Kumar Gupta,[a] Daniel Strand,[a] Arkady Yartsev,*[b] Reiner Lomoth,*[c] Petter Persson,*[d] Kenneth Wärnmark*[a]

[+] = shared authorship, * = Corresponding author

[a]Centre for Analysis and Synthesis, Department of Chemistry, Lund University, Box 124, SE-22100 Lund, Sweden

[b]Chemical Physics Division, Department of Chemistry, Lund University, Box 124, SE-22100 Lund, Sweden

[c]Department of Chemistry – Ångström Laboratory, Uppsala University, Box 523, SE-751 20 Uppsala, Sweden

[d]Theoretical Chemistry Division, Department of Chemistry, Lund University, Box 124, SE-22100 Lund, Sweden

[e]Department of Chemistry, University of Copenhagen, Universitetsparken 5, DK-2100 Copenhagen, Denmark

[f]Department of Physics – Ångström Laboratory, Uppsala University, Box 523, SE-751 20 Uppsala, Sweden

*A.Y.: E-mail: arkady.yartsev@chemphys.lu.se.

*R.L.: E-mail: reiner.lomoth@kemi.uu.se.

*P.P.: E-mail: petter.persson@teokem.lu.se.

*K.W.: E-mail: kenneth.warnmark@chem.lu.se.

Table of Contents

S1. Experimental **3**

S1.1 General experimental details 3

S1.2 Tris[1,1'-methylenebis(3-methylimidazol-1-ylidene)]iron(III) tris(hexafluorophosphate) [Fe(III)(mbmi)$_3$](PF$_6$)$_3$ 3

S1.3 Bis[1,1'-methylenebis(3-methylimidazol-1-ylidene)](2,2'-bipyridine)iron(II) bis(hexafluorophosphate) [Fe(II)(mbmi)$_2$(bpy)](PF$_6$)$_2$ 4

S2 ^{1}H and ^{13}C NMR Spectra **5**

S3 HR-MS spectra **7**

S4 Single Crystal X-ray Diffraction **8**

S5 Mössbauer spectroscopy **15**

S6 Magnetic susceptibility and magnetization measurements **16**

S7 Electron Paramagnetic Resonance Spectroscopy **16**

S8 Electro- and spectroelectrochemistry **17**

S9 Steady State Absorption and Emission Spectroscopy **18**

S10 Quantum yield measurement of [Fe(III)(mbmi)$_3$](PF$_6$)$_3$ **19**

S11 Transient absorption spectroscopy **20**

S12 Quantum Chemistry **20**

S13 References **50**

S1 Experimental

S1.1 General experimental details

^1H and ^{13}C NMR spectra were recorded on a Bruker Avance II 400 MHz NMR spectrometer. Chemical shifts (δ) are reported to the shift-scale calibrated with the residual NMR solvent; CD_3CN (1.94 ppm for ^1H NMR spectra). Electrospray ionization–high resolution mass spectrometry (ESI–HRMS) was recorded on a Waters Micromass Q-Tof micro mass spectrometer. Melting points of the compounds were measured on a Stuart Scientific Melting Point Apparatus-SMP3. Elemental analyses were performed by Mikroanalytisches Laboratorium KOLBE (Mülheim an der Ruhr, Germany).

Potassium *tert*-butoxide (1 M solution in THF) was purchased from Aldrich. Anhydrous ferrous bromide ($FeBr_2$) and potassium hexafluorophosphate (KPF_6) were purchased from Acros. 1,1'-Methylenebis(3-methyl-1*H*-imidazol-3-ium) dihexafluorophosphate ($[mbmiH_2](PF_6)_2$)[S1] and $Fe(bpy)Cl_2$[S2] were synthesized using literature methods. THF (Honeywell) was dried over Na/benzophenone and was subsequently distilled under argon prior to use. Anhydrous CH_3CN and diethyl ether was obtained from a PureSolv PSM-768 and Braun SPS-800 system respectively.

S1.2 Tris[1,1'-methylenebis(3-methylimidazol-1-ylidene)]iron(III) tris(hexafluorophosphate) $[Fe(III)(mbmi)_3](PF_6)_3$

1,1'-Methylenebis(3-methyl-1*H*-imidazol-3-ium) dihexafluorophosphate (0.351 g, 0.750 mmol) was vacuum-dried at 80 °C overnight in a Schlenk tube. Dry THF (30 mL) was charged under N_2. The suspension was then cooled to −78 °C and *t*-BuOK (1.0 M in THF, 1.55 mL, 1.55 mmol) was added dropwise and the reaction mixture was stirred for 30 min at −78 °C. The cooling bath was removed and anhydrous $FeBr_2$ (0.054 g, 0.25 mmol) in 20 mL dry THF was injected to the Schlenk tube containing the in situ generated carbene ligand solution with a syringe before the mixture warmed to room temperature under N_2. The resulting solution was stirred in dark under N_2 at room temperature for 24 hrs. The solvent was evaporated and resulting residue was treated with aqueous solution of NH_4PF_6 (0.326 g, 2.00 mmol) and extracted with CH_3CN (2 x 25 mL). The collected extraction was filtered and washed with CH_3CN. The resulting filtrate was evaporated to dryness and the resulting dark-red residue was washed with water and dried in *vacuo*. The resulting dark-red residue was dissolved in 10 mL of acetonitrile and the product was precipitated by addition of dry diethyl ether (100 mL). The dark-red precipitate was dissolved in a minimum amount of CH_3CN. The solution was

filtered through a syringe filter w/ 0.2 µm PTFE membrane, and the compound was purified on a Bio-Beads S-X1 size-exclusion chromatography column (4 x 125 cm, 1:1 MeCN:Toluene). The product containing fraction was evaporated to dryness under vacuum and the resulting residue was recrystallized from dry CH_3CN via slow diffusion of dry diethyl ether in the dark to yield dark-red crystals (0.077 g, Yield: 30%) tris[1,1'-methylenebis(3-methylimidazol-1-ylidene)]iron(III) tris(hexafluorophosphate).

^1H NMR (400 MHz, CD_3CN, 15 mM): δ(ppm) 33.92 (s, 6H), 19.03 (s, 2H), 6.60 (s, 2H), 8.54 (s, 2H), 3.60 (s, 6H), 3.21 (s, 3H), 2.30 (s, 3H), -7.14 -7.63 (m, 2H), -10.14 (s, 2H), -13.47 (s, 6H), -18.53 (s, 2H). MP: 345. ESI-HRMS calc. for $[(C_{27}H_{36}N_{12}Fe+2PF_6)-(PF_6)]^+$ 874.1819, found 874.1830. Elemental analysis: calc. for $[Fe(II)(mbmi)_3](PF_6)_3$ ($C_{27}H_{36}F_{18}FeN_{12}P_3$) C, 31.81; H, 3.56; N, 16.49; found C, 31.70; H, 3.54; N, 16.43.

S1.3 Bis[1,1'-methylenebis(3-methylimidazol-1-ylidene)](2,2'-bipyridine)iron(II) bis(hexafluorophosphate) $[Fe(II)(mbmi)_2(bpy)](PF_6)_2$

1,1'-Methylenebis(3-methyl-1*H*-imidazol-3-ium) dihexafluorophosphate (0.468 g, 1.00 mmol) and $Fe(bpy)Cl_2$ (0.142 g, 0.5 mmol) were dried in a 100-mL Schlenk tube under vacuum. Anhydrous THF (25 mL) was charged inside under nitrogen and the mixture was cooled down to -78°C. *t*-BuOK (1 M in THF, 2.05 mL, 2.05 mmol) was added using a syringe. The reaction mixture was warmed to room temperature and stirred overnight under N_2 atmosphere in the dark. After completion, the resulting precipitate was collected by filtration using glass sinter (porosity #4) and washed with THF to give product as dark green powder. The green powder was extracted with dry CH_3CN and recrystallized from CH_3CN via slow diffusion of diethyl ether to get dark green fine crystalline powder (0.250 g, Yield 48%) of bis[1,1'-methylenebis(3-methylimidazol-1-ylidene)](2,2'-bipyridine)iron(II) bis(hexafluorophosphate). ^1H NMR (400 MHz, CD_3CN, 10 mM): δ(ppm) 8.46-8.44 (m, 2H, bpy), 8.00-7.95 (m, 2H, bpy), 7.76-7.75 (m, 2H, bpy), 7.29 (m, 2H, im), 7.29-7.25 (m, 2H, bpy), 7.24-7.19 (m, 4H, im), 6.76-6.75 (m, 2H, im), 5.81 (m, 2H, im-CH_2), 3.85-3.81 (m, 2H, im-CH_2), 2.93 (s, 6H, im-methyl), 2.41 (s, 6H, im-methyl). ^{13}C NMR (100 MHz, CD_3CN, 25 mM) δ(ppm) 197.51, 196.37, 160.81, 155.45, 135.95, 126.44, 125.66, 123.08, 122.96, 122.84, 62.02, 36.27, 36.10. MP: 289 °C. ESI+HRMS calc. for $[(C_{28}H_{32}N_{10}Fe)(PF_6)]^+$ 709.1803, found 709.1822. Elemental analysis: calc. for $[Fe(II)(mbmi)_2(bpy)](PF_6)_2$ ($C_{28}H_{32}F_{12}FeN_{10}P_2$) C, 39.36; H, 3.78; N, 16.39; found C, 39.23; H, 3.72; N, 16.24.

S2 ^{1}H and ^{13}C NMR Spectra

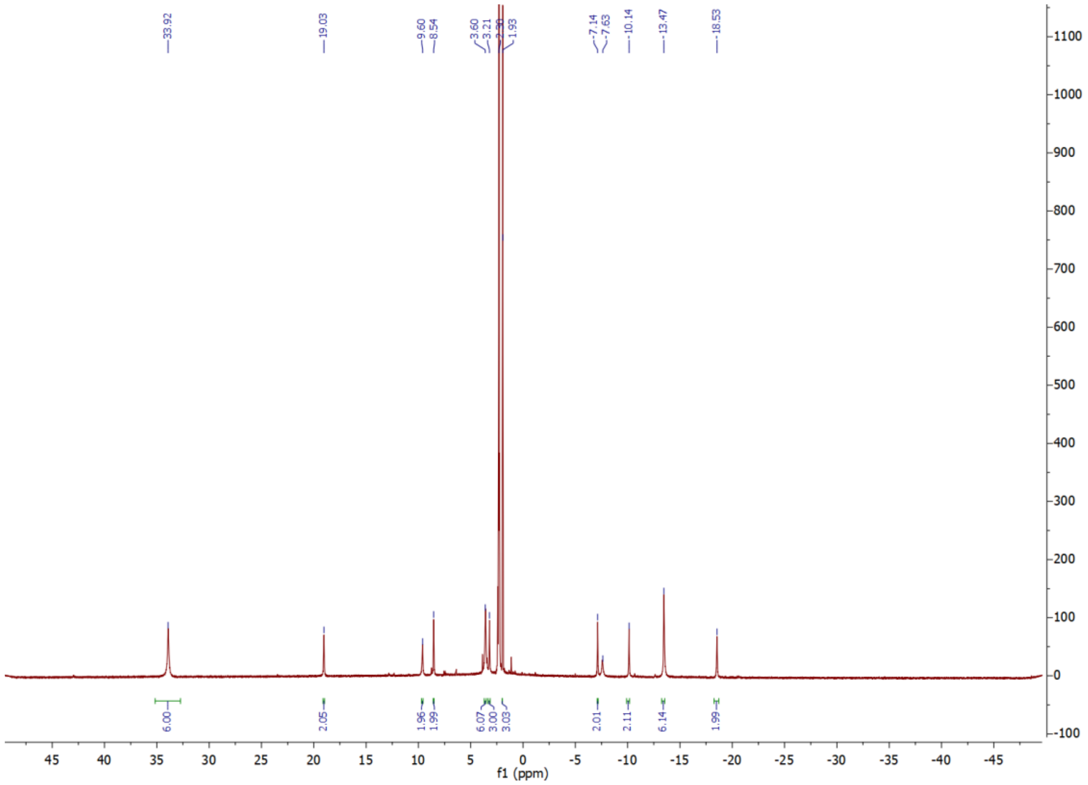

Figure S1. *^{1}H NMR spectrum of $[Fe(III)(mbmi)_3](PF_6)_3$ (15 mM) in CD_3CN.*

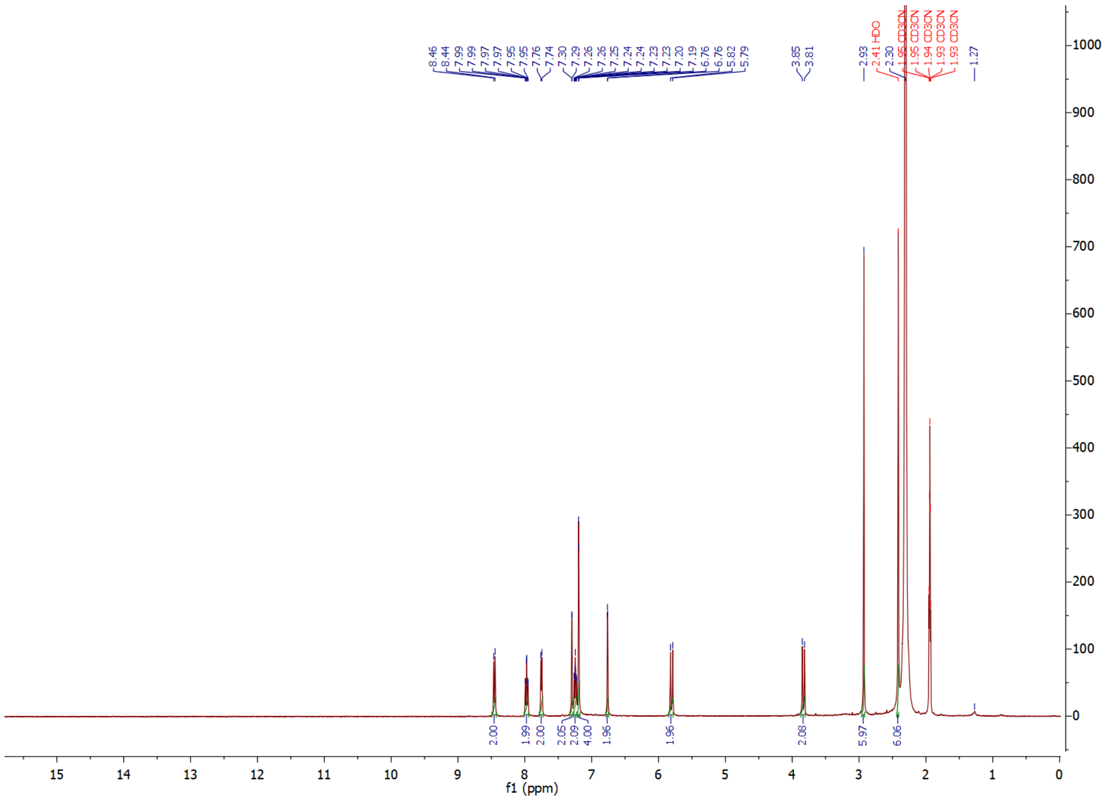

Figure S2. *^{1}H NMR spectrum of $[Fe(II)(mbmi)_2(bpy)](PF_6)_2$ (10 mM) in CD_3CN.*

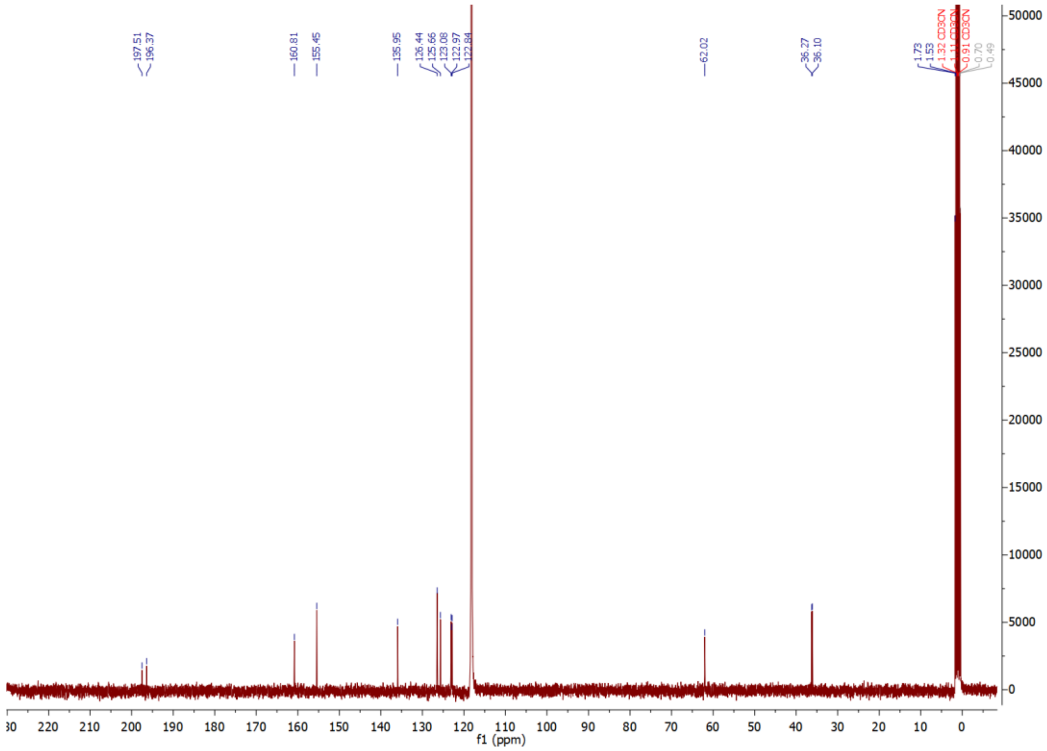

Figure S3. *^{13}C NMR spectrum of $[Fe(II)(mbmi)_2(bpy)](PF_6)_2$ (25 mM) in CD_3CN.*

S3 HR-MS spectra

Single Mass Analysis
Tolerance = 2.0 mDa / DBE: min = -0.5, max = 100.0
Element prediction: Off
Number of isotope peaks used for i-FIT = 3
Monoisotopic Mass, Odd Electron Ions
242 formula(e) evaluated with 1 results within limits (all results (up to 1000) for each mass)
Elements Used:
C: 0-30 H: 0-50 N: 10-15 F: 12-12 Na: 0-1 P: 0-3
56Fe: 0-1

Mass	Calc. Mass	mDa	PPM	DBE	Formula		Fit Conf %	C	H	N	F	Na	P	56Fe
874.1830	874.1819	1.1	1.3	11.0	C27 H36 N12 F12 P2 56Fe		2n.n/a	27	36	12	12		2	1

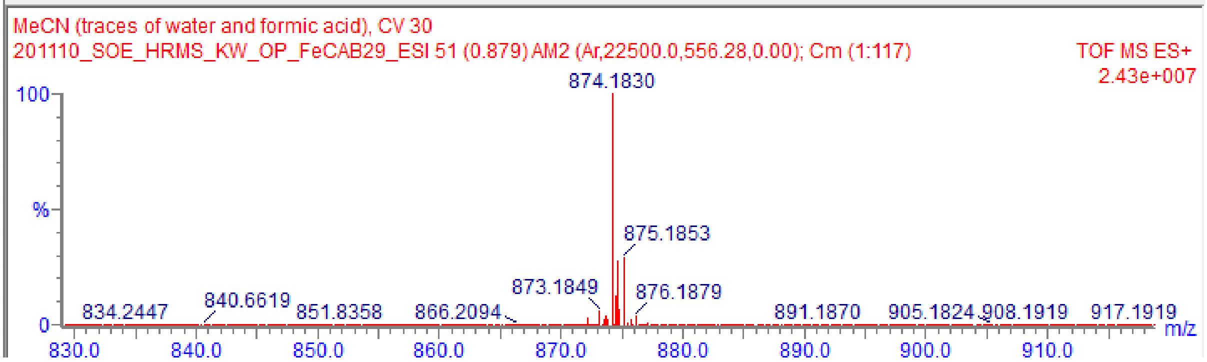

Figure S4. *ES HR-MS spectrum of [Fe(II)(mbmi)$_3$](PF$_6$)$_3$.*

Single Mass Analysis
Tolerance = 2.0 mDa / DBE: min = -0.5, max = 100.0
Element prediction: Off
Number of isotope peaks used for i-FIT = 3
Monoisotopic Mass, Even Electron Ions
167 formula(e) evaluated with 2 results within limits (all results (up to 1000) for each mass)
Elements Used:
C: 0-30 H: 0-50 N: 10-15 F: 6-6 P: 0-3 56Fe: 0-1

Mass	Calc. Mass	mDa	PPM	DBE	Formula	i-F...	Fit Conf %	C	H	N	F	P	56Fe
709.1822	709.1809	1.3	1.8	6.5	C20 H38 N12 F6 P3 56Fe	8...	0.03	20	38	12	6	3	1
	709.1803	1.9	2.7	15.5	C28 H32 N10 F6 P 56Fe	8...	99.97	28	32	10	6	1	1

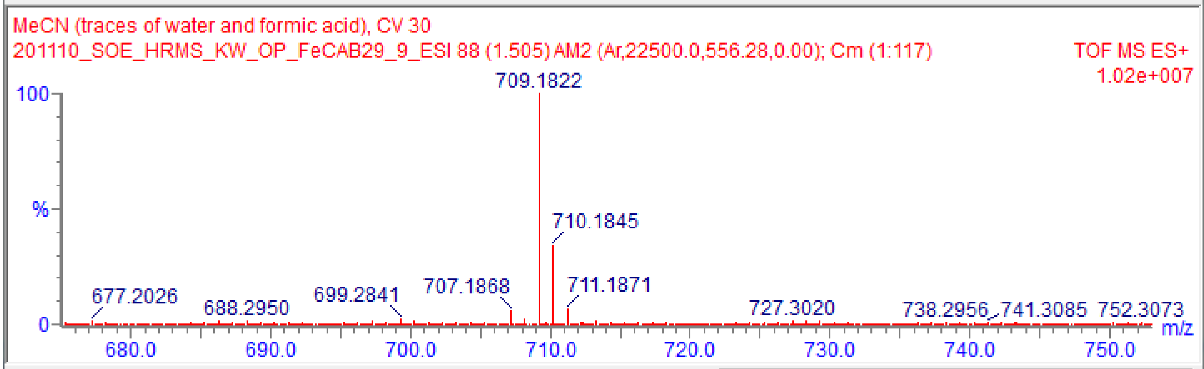

Figure S5. *ES HR-MS spectrum of [Fe(II)(mbmi)$_2$(bpy)](PF$_6$)$_2$.*

S4 Single Crystal X-ray Diffraction

All SC-XRD measurements were performed using graphite-monochromatized Mo Kα radiation (λ = 0.71073 Å) using the Agilent Xcalibur Sapphire3 diffractometer high-brilliance IμS radiation source. Data collections were performed at 293 K for [Fe(II)(mbmi)$_2$(bpy)](PF$_6$)$_2$, and 110 K for [Fe(III)(mbmi)$_3$](PF$_6$)$_3$. Absorption was corrected for using multi-scan empirical absorption correction with spherical harmonics as implemented in the SCALE3 ABSPACK scaling algorithm.[S3] The structure was solved by direct methods and refined by full-matrix least-squares techniques against F2 using all data (SHELXT, SHELXS).[S4, S5] All non-hydrogen atoms were refined with anisotropic displacement parameters if not stated otherwise. Hydrogen atoms constrained in geometric positions to their parent atoms using OLEX2 software.[S6]

Table S1. Structural bond lengths (Fe-L) and bond angles (L-Fe-L) of [Fe(III)(mbmi)$_3$](PF$_6$)$_3$ and [Fe(II)(mbmi)$_2$(bpy)](PF$_6$)$_2$ compared to previously reported [Fe(III)(btz)$_3$](PF$_6$)$_3$[S8] and [Fe(II)(btz)$_2$(bpy)](PF$_6$)$_2$,[S7] respectively.

**Complex**	[Fe(III)(btz)$_3$](PF$_6$)$_3$[S8]	[Fe(III)(mbmi)$_3$](PF$_6$)$_3$	[Fe(II)(btz)$_2$(bpy)](PF$_6$)$_2$[S7]	[Fe(II)(mbmi)$_2$(bpy)](PF$_6$)$_2$
Fe–C (Å)	1.944, 1.968, 1.978	2.010, 2.010, 2.042, 2.043, 2.045, 2.060	1.964, 1.968, 1.995, 2.019	1.951, 1.961, 2.005, 2.012
Fe–N (Å)			1.995, 2.001	1.998, 2.000
C–Fe–C (cis) (°) intraligand (bite angle)	79.24	85.45, 85.69, 89.58	79.29, 79.98	85.50, 85.87
C–Fe–C (cis) (°) interligand	80.71, 100.03, 100.03	82.42, 82.44, 84.77, 84.61, 90.78, 91.15, 91.54, 107.52, 107.63	86.97, 94.01, 96.51	86.50, 87.78, 91.45
C-Fe-N (cis) (°)			85.81, 89.94, 93.99, 94.87, 97.66, 100.98	86.62, 88.15, 93.30, 95.78, 100.30, 100.81
N–Fe–N (°)			80.45	79.57
C–Fe–C (trans) (°)	179.05	166.23, 166.72, 166.78	172.65	169.69
C–Fe–N (trans) (°)			172.68, 178.09	170.65, 171.96

To evaluate the geometry of the coordination sphere of [Fe(III)(mbmi)$_3$](PF$_6$)$_3$ and [Fe(II)(mbmi)$_2$(bpy)](PF$_6$)$_2$ in detail, imaginary planes of average position of atoms in each heterocycle (and central C atom of mbmi) of all ligands were drawn. The angle betweentwo planes within one ligand equals the dihedral angle between the two heterocycles at the central C atom of mbmi (center of C2-C2' bond in bpy), shown in Table S2.

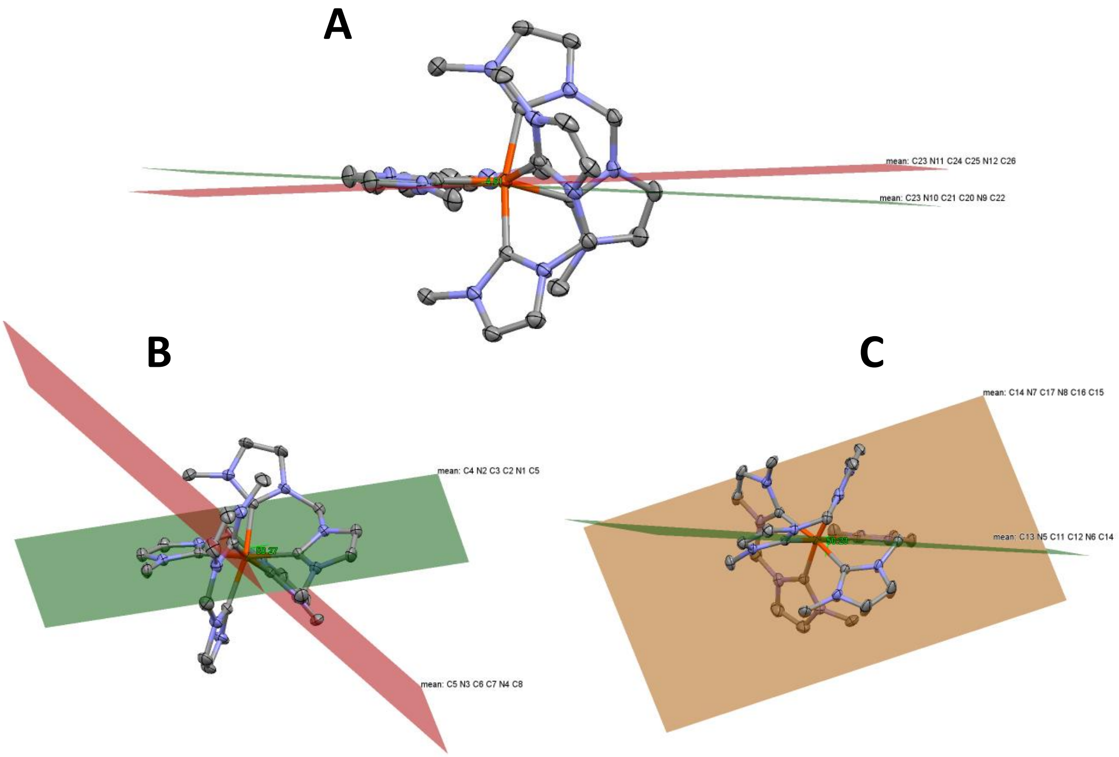

Figure S6. *Imaginary planes drawn through all heterocycles of [Fe(III)(mbmi)$_3$](PF$_6$)$_3$ and dihedral angles of each mbmi ligand shown. A: 4.61°; B: 50.27°; C: 50.23°.*

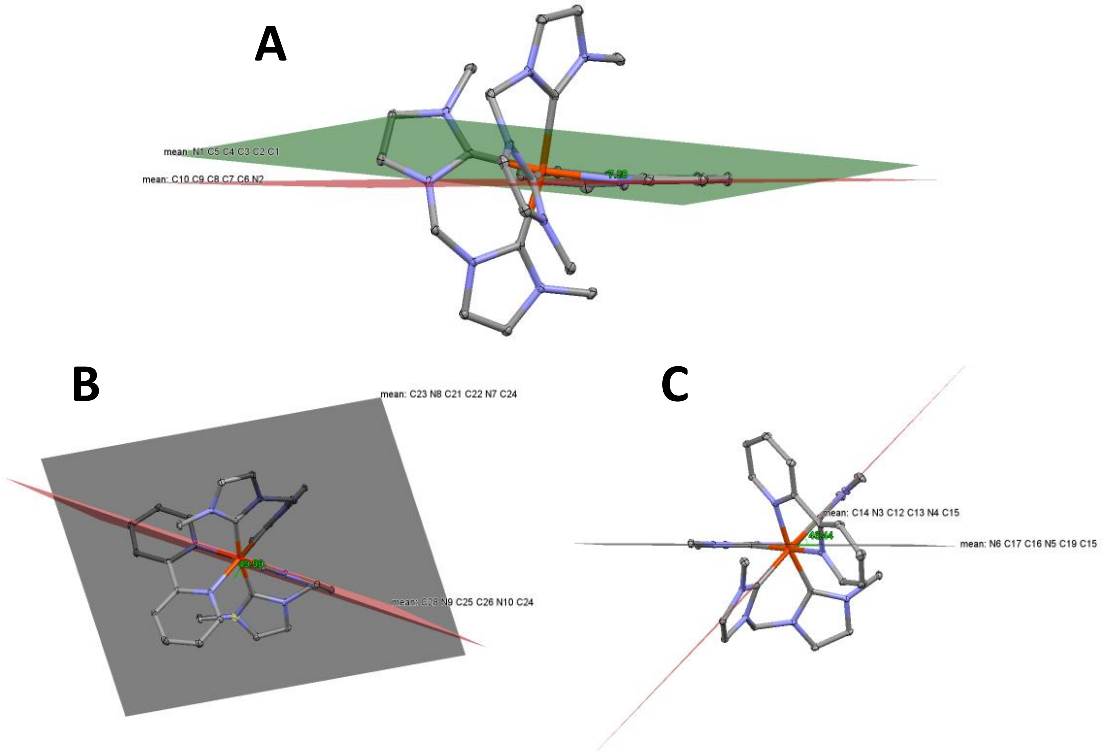

Figure S7. *Imaginary planes drawn through all heterocycles of [Fe(II)(mbmi)$_2$(bpy)](PF$_6$)$_2$ and dihedral angles of each ligand shown. A (bpy): 7.28°; B (mbmi): 49.99°; C: 46.44°.*

Table S2. *Dihedral angles (φ) between the heterocycles of each bidentate ligand of [Fe(III)(mbmi)$_3$](PF$_6$)$_3$ and [Fe(II)(mbmi)$_2$(bpy)](PF$_6$)$_2$ compared to previously reported [Fe(III)(btz)$_3$](PF$_6$)$_3$*[S8] *and [Fe(II)(btz)$_2$(bpy)](PF$_6$)$_2$,*[S8] *respectively.*

	[Fe(III)(btz)$_3$] (PF$_6$)$_3$[S8]	[Fe(III)(mbmi)$_3$] (PF$_6$)$_3$	[Fe(II)(btz)$_2$(bpy)] (PF$_6$)$_2$[S7]	[Fe(II)(mbmi)$_2$ (bpy)](PF$_6$)$_2$
φ^1 (mbmi/btz/bpy)	3.23° (btz)	4.61° (mbmi)	4.25° (bpy)	7.28° (bpy)
φ^2 (mbmi/btz)	3.23° (btz)	50.27° (mbmi)	5.37° (btz)	49.99° (mbmi)
φ^3 (mbmi/btz)	3.23° (btz)	50.23° (mbmi)	7.36° (btz)	46.44° (mbmi)

Table S3. *Bond lengths and angles for [Fe(II)(mbmi)$_2$(bpy)](PF$_6$)$_2$ and [Fe(II)(mbmi)$_3$](PF$_6$)$_3$.*

Compound	Bond lengths (Å)	Bond angles (°)
[Fe(III)(mbmi)$_3$](PF$_6$)$_3$	C(1)-N(1): 1.468(5)	N(1)-C(1)-H(1C): 109.5
	C(3)-N(2): 1.388(5)	C(3)-C(2)-N(1): 108.3(3)
	C(4)-N(1): 1.365(4)	N(1)-C(4)-Fe(1): 133.7(3)
	C(4)-N(2): 1.363(5)	N(2)-C(4)-N(1): 103.6(3)
	C(4)-Fe(1): 2.042(4)	N(2)-C(4)-Fe(1): 122.6(2)
	C(5)-N(3): 1.456(5)	N(3)-C(5)-N(2): 109.8(3)
	C(6)-N(3): 1.400(5)	C(7)-C(6)-N(3): 106.1(4)
	C(7)-N(4): 1.391(6)	C(6)-C(7)-N(4): 107.8(4)
	C(8)-N(3): 1.356(5)	N(3)-C(8)-N(4): 103.2(3)
	C(8)-N(4): 1.374(5)	N(3)-C(8)-Fe(1): 124.9(3)
	C(8)-Fe(1): 2.012(4)	N(4)-C(8)-Fe(1): 131.8(3)
	C(9)-N(4): 1.472(5)	N(5)-C(13)-N(6): 103.4(3)
	C(10)-N(5): 1.464(5)	N(5)-C(13)-Fe(1): 134.4(3)
	C(11)-N(5): 1.394(5)	N(6)-C(13)-Fe(1): 121.9(3)
	C(13)-N(6): 1.375(5)	N(7)-C(17)-Fe(1): 124.8(3)
	C(13)-Fe(1): 2.059(4)	N(8)-C(17)-N(7): 103.6(3)
	C(14)-N(6): 1.448(5)	N(8)-C(17)-Fe(1): 131.4(3)
	C(14)-N(7): 1.464(5)	N(9)-C(22)-Fe(1): 130.8(3)
	C(16)-N(8): 1.404(5)	N(10)-C(22)-N(9): 103.3(3)
	C(17)-N(7): 1.361(5)	N(10)-C(22)-Fe(1): 125.9(3)
	C(17)-N(8): 1.366(5)	N(11)-C(26)-Fe(1): 126.7(3)
	C(17)-Fe(1): 2.011(4)	N(12)-C(26)-Fe(1): 130.9(3)
	C(18)-N(8): 1.483(5)	C(4)-Fe(1)-C(13): 166.30(16)
	C(21)-N(10): 1.401(6)	C(4)-Fe(1)-C(22): 82.38(15)
	C(22)-N(9): 1.375(5)	C(4)-Fe(1)-C(26): 107.53(15)

	C(22)-N(10): 1.366(5)<br>C(22)-Fe(1): 2.046(4)<br>C(23)-N(10): 1.454(6)<br>C(23)-N(11): 1.451(6)<br>C(24)-N(11): 1.387(6)<br>C(26)-Fe(1): 2.041(4)<br>C(27)-N(12): 1.475(6)	C(8)-Fe(1)-C(4): 85.58(14)<br>C(8)-Fe(1)-C(13): 84.78(15)<br>C(8)-Fe(1)-C(22): 91.17(15)<br>C(8)-Fe(1)-C(26): 166.84(15)<br>C(17)-Fe(1)-C(4): 84.75(14)<br>C(17)-Fe(1)-C(8): 90.82(15)<br>C(17)-Fe(1)-C(13): 85.67(15)<br>C(17)-Fe(1)-C(22): 166.78(14)<br>C(17)-Fe(1)-C(26): 91.48(16)<br>C(22)-Fe(1)-C(13): 107.52(17)<br>C(26)-Fe(1)-C(13): 82.47(15)<br>C(26)-Fe(1)-C(22): 89.55(16)
[Fe(II)(mbmi)$_2$(bpy)](PF$_6$)$_2$	C(5)-N(1): 1.366(4)<br>C(6)-N(2): 1.357(4)<br>C(10)-N(2): 1.347(4)<br>C(12)-N(3): 1.380(4)<br>C(13)-N(4): 1.377(4)<br>C(14)-N(4): 1.365(4)<br>C(14)-Fe(1): 2.012(3)<br>C(15)-N(4): 1.445(4)<br>C(16)-N(5): 1.392(4)<br>C(18)-N(6): 1.458(4)<br>C(19)-N(5): 1.360(4)<br>C(19)-N(6): 1.376(3)<br>C(19)-Fe(1): 1.962(3)<br>C(23)-N(7): 1.373(4)<br>C(23)-Fe(1): 2.005(3)<br>C(24)-N(7): 1.449(4)<br>C(24)-N(9): 1.445(4)	N(1)-C(1)-C(2): 122.9(3)<br>N(1)-C(5)-C(4): 122.0(3)<br>N(1)-C(5)-C(6): 113.4(3)<br>N(2)-C(6)-C(5): 113.9(3)<br>N(2)-C(6)-C(7): 122.7(3)<br>N(2)-C(10)-C(9): 123.5(3)<br>C(12)-C(13)-N(4): 105.7(3)<br>N(3)-C(14)-Fe(1): 135.7(2)<br>N(4)-C(14)-N(3): 101.8(3)<br>N(4)-C(14)-Fe(1): 122.6(2)<br>N(5)-C(19)-N(6): 102.5(2)<br>N(5)-C(19)-Fe(1): 125.0(2)<br>N(6)-C(19)-Fe(1): 132.3(2)<br>N(8)-C(23)-Fe(1): 135.0(2)<br>C(26)-C(25)-N(9): 106.1(3)<br>N(9)-C(28)-N(10): 102.3(3)<br>N(9)-C(28)-Fe(1): 125.1(2)

	C(25)-N(9): 1.384(4)	N(10)-C(28)-Fe(1): 132.6(2)
	C(26)-N(10): 1.382(4)	C(1)-N(1)-C(5): 117.0(3)
	C(27)-N(10): 1.473(4)	C(1)-N(1)-Fe(1): 126.1(2)
	C(28)-N(9): 1.366(4)	C(5)-N(1)-Fe(1): 116.4(2)
	C(28)-N(10): 1.366(4)	C(6)-N(2)-Fe(1): 116.3(2)
	C(28)-Fe(1): 1.950(3)	C(10)-N(2)-Fe(1): 126.3(2)
	N(1)-Fe(1): 1.998(3)	C(19)-Fe(1)-C(14): 85.53(13)
	N(2)-Fe(1): 2.000(3)	C(19)-Fe(1)-C(23): 87.70(12)
	N(101)-O(101): 1.183(6)	C(19)-Fe(1)-N(1): 172.02(12)
	N(101)-O(102): 1.213(5)	C(19)-Fe(1)-N(2): 95.78(11)
	N(102)-O(103): 1.242(6)	C(23)-Fe(1)-C(14): 169.64(13)
	N(102)-O(104): 1.145(5)	C(28)-Fe(1)-C(14): 86.50(11)
		C(28)-Fe(1)-C(19): 91.44(12)
		C(28)-Fe(1)-C(23): 85.86(11)
		C(28)-Fe(1)-N(1): 93.85(11)
		C(28)-Fe(1)-N(2): 170.65(11)
		N(1)-Fe(1)-C(14): 100.75(11)
		N(1)-Fe(1)-C(23): 86.73 (11)
		N(1)-Fe(1)-N(2): 79.63(10)
		N(2)-Fe(1)-C(14): 88.19(11)
		N(2)-Fe(1)-C(23): 100.30(12)

Table S4. *Crystal data and structure refinement for [Fe(III)(mbmi)$_3$](PF$_6$)$_3$.*

Identification code	[Fe(III)(mbmi)$_3$](PF$_6$)$_3$
CCDC No.	2040117
Empirical formula	$C_{29}H_{39}F_{18}FeN_{13}P_3$
Formula weight	1060.49
Temperature	110(2) K
Wavelength	0.71073 Å
Crystal system	Monoclinic
Space group	*P 2(1)/c*

Unit cell dimensions	*a* = 12.8072(9) Å	α = 90°.
	b = 15.4052(15) Å	β = 97.881(7)°.
	c = 22.6074(17) Å	γ = 90°.
Volume	4418.3(6) $Å^3$	
Z	4	
Density (calculated)	1.594 Mg/m^3	
Absorption coefficient	0.567 mm^{-1}	
F(000)	2148	
Crystal size	0.5 x 0.5 x 0.3 mm^3	
Theta range for data collection	2.586 to 28.843°.	
Index ranges	-17<=h<=15, -19<=k<=19, -29<=l<=29	
Reflections collected	37728	
Independent reflections	10091 [*R*(int) = 0.0534]	
Completeness to theta = 25.242°	99.9 %	
Absorption correction	Semi-empirical from equivalents	
Max. and min. transmission	1.00000 and 0.85072	
Refinement method	Full-matrix least-squares on F^2	
Data / restraints / parameters	10091 / 36 / 713	
Goodness-of-fit on F^2	1.015	
Final *R* indices [*I*>2sigma(I)]	*R1* = 0.0717, *wR2* = 0.1554	
R indices (all data)	*R1* = 0.1246, *wR2* = 0.1848	
Largest diff. peak and hole	0.563 and -0.296 e.$Å^{-3}$	

Table S5. *Crystal data and structure refinement for [Fe(II)(mbmi)$_2$(bpy)](PF$_6$)$_2$.*

Identification code	[Fe(II)(mbmi)$_2$(bpy)](PF$_6$)$_2$	
CCDC No.	2040116	
Empirical formula	$C_{30}H_{38}F_{12}FeN_{12}O_4P_2$	
Formula weight	976.51	
Temperature	293(2) K	
Wavelength	0.71073 Å	
Crystal system	Triclinic	
Space group	P-1	
Unit cell dimensions	*a* = 10.9186(5) Å	α = 78.144(4)°.
	b = 11.1349(5) Å	β = 88.763(4)°.
	c = 16.7152(8) Å	γ = 81.006(4)°.
Volume	1964.27(16) $Å^3$	
Z	2	
Density (calculated)	1.651 Mg/m^3	
Absorption coefficient	0.576 mm^{-1}	
F(000)	996	
Crystal size	0.1 x 0.1 x 0.1 mm^3	
Theta range for data collection	3.423 to 29.355°.	

Index ranges	-14<=h<=14, -14<=k<=13, -22<=l<=21
Reflections collected	21934
Independent reflections	9186 [*R*(int) = 0.0544]
Completeness to theta = 25.242°	99.7 %
Absorption correction	Semi-empirical from equivalents
Max. and min. transmission	1.00000 and 0.94896
Refinement method	Full-matrix least-squares on F^2
Data / restraints / parameters	9186 / 98 / 593
Goodness-of-fit on F^2	1.037
Final *R* indices [*I*>2sigma(I)]	*R1* = 0.0632, *wR2* = 0.1194
R indices (all data)	*R1* = 0.0962, *wR2* = 0.1355
Largest diff. peak and hole	1.078 and -0.525 e.Å^{-3}

S5 Mössbauer spectroscopy

Mössbauer measurements were carried out in an Oxford Instrument flow cryostat at temperatures between 85 K and 295 K using a ^{57}CoRh source. The powder samples were mixed with BN to form absorbers with a concentration of 20mg/cm^2. Calibration spectra were recorded from an iron metal foil. The resulting spectra were analyzed using a least square Mössbauer fitting program.

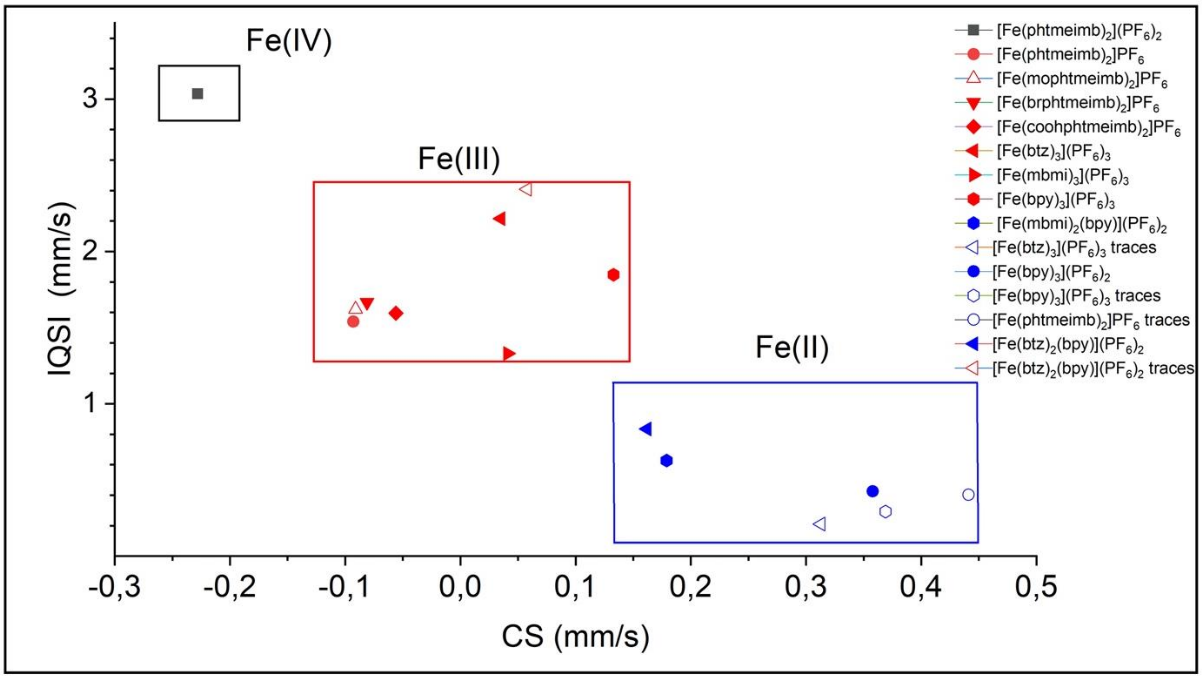

Figure S8. *Electric quadrupole splittings |QS| and center shifts CS at 80K, versus natural Fe at room temperature, for [Fe(mbmi)$_3$](PF$_6$)$_3$, [Fe(mbmi)$_2$(bpy)](PF$_6$)$_2$ and previously reported similar complexes.*[S7-S10]

S6 Magnetic susceptibility and magnetization measurements

The magnetic data were acquired on a Quantum-Design MPMS-XL SQUID magnetometer. Susceptibility data were acquired in a static field of 1.0 KOe. Magnetization data were obtained with selected fields from 1 to 50 KOe at T=2-10 K in 1K intervals. The polycrystalline samples were measured on a compacted powder sample in a polycarbonate capsule. Data were corrected empirically for TIP and the diamagnetic contribution to the sample moment from the sample holder and sample was corrected through background measurements and Pascal constants, respectively.

S7 Electron Paramagnetic Resonance Spectroscopy

X-band EPR measurements were performed on a BrukerELEXYS E500 spectrometer equipped with a SuperX EPR049 microwave bridge and a cylindrical TE_{011} ER 4122SHQE cavity. The temperature was controlled using an Oxford Instruments ESR 900 flow cryostat. The microwave frequency was operated at 9.4 GHz. Microwave power of 2 mW; modulation frequency of 100 kHz at the amplitude of 1 mT and temperature at 15 K were applied for all spectral recordings. Samples of [Fe(II)(mbmi)$_3$](PF$_6$)$_3$ and [Fe(III)(mbmi)$_3$(PF$_6$)$_3$ were dissolved in argon purged, dry acetonitrile to a concentration of 2 mM and 6 mM respectively and transferred into EPR tubes under dim-light. All samples were stored in liquid nitrogen and in darkness before EPR examination.

[Fe(II)(mbmi)$_2$(bpy)](PF$_6$)$_2$ was not expected to show EPR spectra. However, the Fe(II) complex did show signals of unknown origin (marked with asterisks) around the g-2 region (Fig. S8a). Based on the poor S/N ratio, and low signal intensity, most likely some impurities were culpable. On the other hand, we had expected [Fe(III)(mbmi)$_3$(PF$_6$)$_3$ sample to show EPR spectrum (see main text). The measured spectrum (Fig. S8b) showed many features, though at very low intensity. Besides, several of them shared same character with those in the spectrum for [Fe(II)(mbmi)$_2$(bpy)](PF$_6$)$_2$ (marked with asterisks in Fig. S8a), deemed inherited from the synthesis processes. Apart from these, some new signals, for example at g 2.3 and 1.8 were found, which may be assigned to spin ½ species. However, the very low signal intensity represents only a trace part of the spin quantity expected, and thus can only be assigned to other unknown Fe(III) species than the main complex [Fe(III)(mbmi)$_3$(PF$_6$)$_3$.

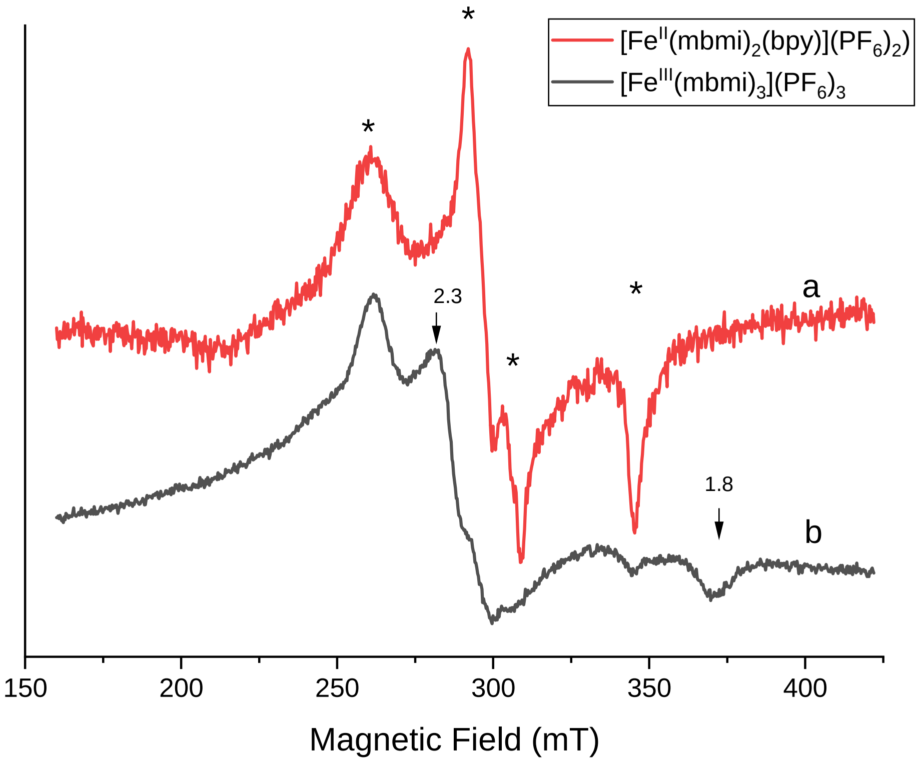

Figure S9. *EPR spectra of [Fe(II)(mbmi)$_2$(bpy)](PF$_6$)$_2$ (**a** - red) and [Fe(III)(mbmi)$_3$(PF$_6$)$_3$ (**b** - black).*

S8 Electro- and spectroelectrochemistry

Electrochemical and spectro-electrochemical measurements were carried out in a setup previously described[7] which utilized a custom-made quartz cell (optical path length: 1 mm) and a standard three electrode setup consisting of a working (1 mm dia., glassy carbon, CH Instruments), counter (platinum rod) and reference electrode (0.01 M Ag^+/Ag). Spectroscopic grade acetonitrile dried for 48 h over 3 Å activated molecular sieves was used as solvent, together with 0.1 M tetrabutylammonium hexafluorophosphate (electrochemical grade, Sigma) dried for 24 h under vacuum at 80°C as supporting electrolyte. Cyclic voltammograms were record at 0.1 V/s, and differential pulse voltammograms with step potential: 5 mV, modulation amplitude: 25 mV, modulation time: 0.05 s, interval time: 0.1 s. UV-Vis spectroelectrochemistry was carried out in the same cell by switching the working electrode to a platinum mesh electrode placed in the 1 mm optical path to facilitate bulk electrolysis, and traces were collected every 5 seconds for 600 seconds (note the figures show every second scan, i.e. every 10 seconds). An Autolab potentiostat (PGSTAT302) was used to control

the three-electrode setup using the GPES 4.9 software, and an Agilent 8453 diode array spectrophotometer was used to record the spectral traces.

S9 Steady State Absorption and Emission Spectroscopy

Linear absorption measurements were carried out on Perkin Elmer Lambda 1050 absorption spectrometer using standard cuvettes (1mm path length; Hellma OS glass) and are corrected for reference measurement using the same cuvette with pristine acetonitrile as reference.

To confirm that the emission is correlated to the LMCT state, an absorption luminescence excitation measurement was performed. The luminescence intensity at the peak of the emission spectrum (650 nm) was recorded for varied excitation wavelength using Horiba Fluorolog 3-21 Spectrofluorometer and standard cuvettes (1 cm optical path length; Hellma OS glass) and front face geometry. The emission intensity (orange line in Fig. S10) reproduces both features of the absorption spectrum (black line in Fig. S10); confirming that the emission is stemming from the lowest energy LMCT absorption.

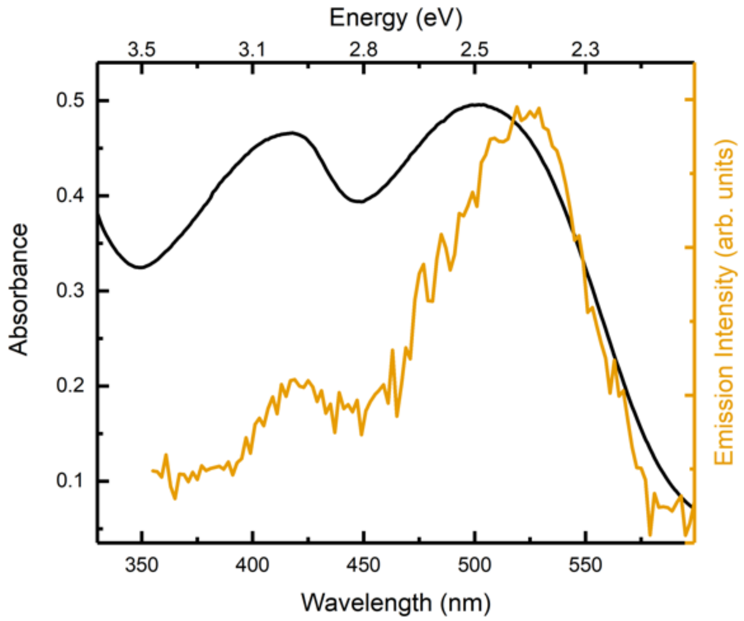

Figure S10. *Absorption (black) and luminescence excitation spectrum (orange) of $[Fe(mbmi)_3](PF_6)_3$ in acetonitrile, recorded at 650 nm.*

S10 Quantum yield measurement of [Fe(III)(mbmi)$_3$](PF$_6$)$_3$

We measured quantum yield of [Fe(mbmi)$_3$](PF$_6$)$_3$ relative to that of [Fe^{III}(phtmeimb)$_2$]PF$_6$, (phtmeimb = [phenyltris(3-methyl-imidazolin-2-ylidene)borate$^-$]), for which the quantum yield was taken from.[7] Agilent 8453 UV-Visible spectrometer was used to measure absorption spectra and Flourolog-3 fluorimeter (Horiba) was used to measure emission spectra. Six different concentrations in anhydrous acetonitrile were measured for both [Fe(mbmi)$_3$](PF$_6$) and the reference sample [Fe^{III}(phtmeimb)$_2$]PF$_6$ with the absorbance ranging from 0.05 to 0.15.

Luminescent quantum yield of [Fe(mbmi)$_3$](PF$_6$) was determined using the following equation:

$$\phi_{[\mathrm{Fe(mbmi)_3](PF_6)}} = \phi_{[\mathrm{Fe^{III}(phtmeimb)_2]PF_6}} \times \frac{Grad_{[\mathrm{Fe(mbmi)_3](PF_6)}}}{Grad_{[\mathrm{Fe^{III}(phtmeimb)_2]PF_6}}}$$

$$= 0.000123 \pm 0.0000084$$

where *Grad*'s are the slopes of integrated fluorescence area under the emission spectra. The values 1-10^{-A} were extracted from absorption spectra at the excitation wavelength 500 nm for each concentration of both samples, as shown in Figure S11.

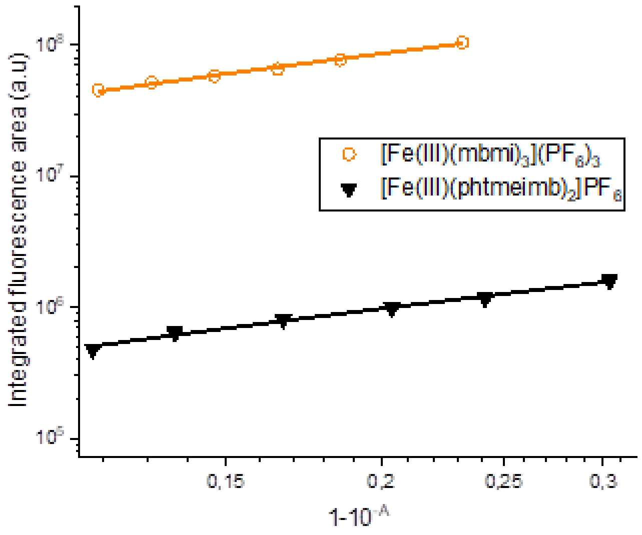

Figure S11. *Integrated fluorescence areas of [Fe(mbmi)$_3$](PF$_6$) (black triangles) and [Fe^{III}(phtmeimb)$_2$]PF$_6$ (orange circles) vs 1-10^{-A} along with the corresponding linear fits.*

S11 Transient absorption spectroscopy

The experimental setup for recording broadband transient absorption spectra was based on a Ti:Sapphire amplified laser system (Spitfire XP Pro, Spectra Physics) operating at a 1 kHz repetition rate, producing ~80 fs pulses centered at 796 nm. The pump beam was tuned by an optical parametric amplifier (TOPAS C, Light Conversion) to excite the sample at various wavelengths with typical fluency not exceeding 3 x 10^{14} photons per pulse per cm^2. A super-continuum white-light was used as probe, generated by focusing NIR signal from a TOPAS C into a 5 mm sapphire plate. The desired timing between excitation and probe pulses was achieved by a computer-controlled delay line (Aerotech, 10 ns). The pump and the probe beams were overlapped on the sample with their relative polarization set to the magic-angle (54.7º) by a Berek polarization compensator placed in the pump beam path. The sample was placed in a quartz 1 mm path length cuvette with an automated sample mover to avoid sample photodamage, which was checked by measuring absorption spectra of the sample before and after each experiment. The probe and reference beams were collimated on the entrance apperture of a prism-based, double-beam spectrograph, and detected by a double diode-array detection system (Pascher Instruments). Correction of the data as well as fitting of the transient absorption datasets was carried out using inhouse analysis software DAFit (Pascher Instruments).

S12 Quantum Chemistry

Quantum chemical calculations were performed with restricted and unrestricted Density Functional of Theory (DFT) using a B3LYP* modification of the standard B3LYP functional with 15 % Hartree-Fock exchange. All calculations were performed with the 6-311G(d) basis set in the Gaussian09 quantum chemical software package.[S11] Geometry optimisations were computed as full relaxations with no imposed symmetry with the numerical ultrafine grid. Solvent effects were accounted for using a polarizable continuum model (PCM) with the dielectric constant of acetonitrile for both optimization and single point calculations. The relaxed structures were assessed to be minima by frequency calculations. A minimum energy path between the ^{3}CT and ^{3}MC excited states in $[Fe(mbmi)_2(bpy)]^{2+}$ complex was estimated by interpolating a set of structures between the two optimized structures and sequentially optimizing all intermediate structures with frozen metal-ligand bonds. Singlet-singlet time

dependent DFT (TD-DFT) vertical excitations were calculated with the same level of theory B3LYP*/6-311G(d) to up to 40 roots for the closed-shell iron complex $[Fe(mbmi)_2(bpy)]^{2+}$.

Table S6. *Ground state geometry specifications for $[Fe(III)(mbmi)_3]^{3+}$ and $[Fe(II)(mbmi)_2(bpy)]^{2+}$. Distances and angles are shown for the iron metal center with carbene carbon and bpy nitrogen.*

**Complex**	$[Fe(III)(mbmi)_3]^{3+}$	$[Fe(II)(mbmi)_2(bpy)]^{2+}$
Fe–C (Å)	2.039, 2.041, 2.056, 2.062, 2.075, 2.079	1.993, 1.993, 2.054, 2.054
Fe–N (Å)	-	2.047, 2.047
C–Fe–C (cis) (°)	86.48, 86.65, 88.84, 92.46	86.37, 87.98, 87.99
C–Fe–C (trans) (°)	167.87, 169.15, 170.19	171.74
N–Fe–N (°)	-	78.90

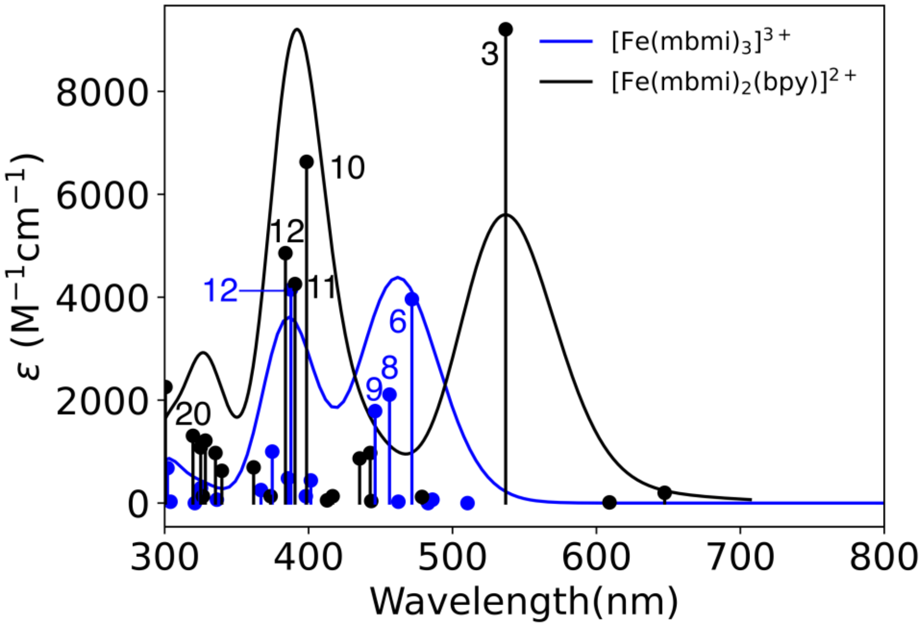

Figure S12. *Calculated UV-vis absorption spectra for [Fe(mbmi)$_2$(bpy)]$^{2+}$ (black) and [Fe(mbmi)$_3$]$^{3+}$ (blue) complexes. Intense vertical excitations are labeled with the corresponding root number. Data collected in Tables S7 and S8.*

Table S7. *TD-DFT singlet-singlet vertical excitations of singlet relaxed [Fe(mbmi)$_2$(bpy)]$^{2+}$ for a selection of roots according to its oscillator strengths (f). The energy for each of the states are represented in eV and corresponding wavelength in nm. Electronic transitions and coefficients are also provided in this table as well as each root assignment.*

Root	E (eV)	λ (nm)	f	Transition/Coefficient		Nature	Assignment
**1**	1.9151	647.41	0.0015	HOMO -> LUMO	0.69373	Fe (d) -> bpy (π)	MLCT
**3**	2.3090	536.95	0.0690	HOMO-2 -> LUMO	0.68585	Fe (d) -> bpy (π)	MLCT
**10**	3.1104	398.61	0.0497	HOMO -> LUMO+2<br>HOMO-2 -> LUMO+1<br>HOMO-1 -> LUMO+2	0.50466<br>0.38579<br>-0.26864	Fe (d) -> bpy (π)	MLCT
**11**	3.1743	390.58	0.0319	HOMO-1 ->LUMO+2	0.60186	Fe(d) -> bpy(π)	MLCT
**12**	3.2294	383.92	0.0364	HOMO-2 ->LUMO+2	0.66824	Fe(d) -> bpy(π)	MLCT

**17**	3.7766	328.30	0.0091	HOMO-1 ->LUMO+4<br>HOMO-4 ->LUMO<br>HOMO-1 ->lUMO+6	0.43589<br>0.31136<br>-0.22187	Fe(d) -> mbmi(π)<br>mbmi(π)->bpy (π)<br>Fe(d)->mbmi(π)/Fe(d)	MLCT
**20**	3.8785	319.67	0.0098	HOMO-2 ->LUMO+4<br>HOMO-2 ->LUMO+6<br>HOMO ->LUMO+3	0.55161<br>0.27583<br>-0.21845	Fe(d) -> mbmi(π)<br>Fe(d)->mbmi(π)/Fe(d)	MLCT
**22**	4.2018	295.07	0.0578	HOMO ->LUMO+8<br>HOMO->LUMO+3	0.42727<br>-0.31156	Fe(d) ->mbmi(π)/Fe(d)	MLCT/MC
**24**	4.3129	287.47	0.2765	HOMO-6 ->LUMO<br>HOMO-7 ->LUMO	0.58054<br>-0.26654	bpy (π) ->bpy (π)	LC
**27**	4.3346	286.03	0.0699	HOMO ->LUMO+7<br>HOMO-1 ->LUMO+7	0.58553<br>0.20276	Fe(d)/mbmi(π) -> mbmi(π)	LC
**28**	4.3585	284.47	0.0485	HOMO-7 ->LUMO<br>HOMO-6 ->LUMO	0.62457<br>0.21894	mbmi(π)/Fe(d) -> bpy(π)	LC
**33**	4.5271	273.87	0.0891	HOMO-1 ->LUMO+7<br>HOMO-2 ->LUMO+5	0.52163<br>-0.34380	Fe(d) -> mbmi(π)	MLCT
**36**	4.6210	268.30	0.0464	HOMO-5 ->LUMO+1	0.67316	mbmi(π)/Fe(d) -> mbmi(π)	LC
**37**	4.6832	264.74	0.0514	HOMO-2 ->LUMO+6<br>HOMO-2 ->LUMO+12<br>HOMO-1 ->LUM0+7	0.40512<br>0.42186<br>-0.20422	Fe(d) -> mbmi(π)/Fe(d)<br>Fe(d) -> mbmi(π)/Fe(d)	MLCT

Table S8. *TD-DFT doublet-doublet vertical excitations of singlet relaxed [Fe(mbmi)$_3$]$^{3+}$ for a selection of roots according to its oscillator strengths (f). The energy for each of the states are represented in eV and corresponding wavelength in nm. Electronic transitions and coefficients are also provided in this table as well as each root assignment.*

**Root**	**E (eV)**	**λ (nm)**	**f**	**Transition/Coefficient**		**Nature**	**Assignment**
**3**	2.4290	510.44	0.0000	αHOMO -> αLUMO<br>βHOMO -> βLUMO<br>βHOMO -> βLUMO+3	0.43370<br>0.47564<br>0.44649	mbmi(π) -> Fe (d)<br>mbmi(π) -> Fe (d)<br>mbmi(π) -> Fe (d)	LMCT
**6**	2.6280	471.79	0.0297	βHOMO-3 -> βLUMO<br>βHOMO-2 -> βLUMO	0.80962<br>0.41589	mbmi(π) -> Fe (d)<br>mbmi(π) -> Fe (d)	LMCT

**8**	2.7176	456.23	0.0158	βHOMO-3 -> βLUMO -0.43566<br>βHOMO-2 -> βLUMO 0.89013	mbmi(π) -> Fe (d)<br>mbmi(π) -> Fe (d)	LMCT
**9**	2.7785	446.23	0.0134	βHOMO-4 -> βLUMO 0.98468	mbmi(π) -> Fe (d)	LMCT
**12**	3.1978	387.72	0.0311	βHOMO-6 -> βLUMO 0.83594	mbmi(π)/Fe(d) -> Fe (d)	LMCT
**21**	4.1063	301.94	0.0051	βHOMO-9 -> βLUMO 0.97305	mbmi(π) -> Fe (d)	LMCT
**22**	4.1269	300.43	0.0050	αHOMO-1 -> αLUMO+2 0.34970<br>βHOMO-14 -> βLUMO -0.29564<br>βHOMO-1 -> βLUMO+1 -0.50064<br>βHOMO-1 -> βLUMO+3 0.51107	Fe(d)/mbmi(π) -> mbmi(π)<br>mbmi(σ)-> Fe(d)<br>Fe(d) -> Fe(d)/mbmi(π)<br>Fe(d) -> mbmi(π)	MLCT
**35**	4.5432	272.90	0.0054	βHOMO-17 -> βLUMO -0.42683<br>βHOMO-15 -> βLUMO -0.39503<br>βHOMO-13 -> βLUMO 0.49979	Fe(d) -> Fe(d)<br>Fe(d)/mbmi(σ) -> Fe(d)<br>mbmi(π) -> Fe(d)	MC/MLCT
**36**	4.5564	272.11	0.0046	βHOMO-17 -> βLUMO 0.32198<br>βHOMO-15 -> βLUMO 0.29679<br>βHOMO-13 -> βLUMO 0.66017	Fe(d) -> Fe(d)<br>Fe(d)/mbmi(σ) -> Fe(d)<br>mbmi(π) -> Fe(d)	MLCT/MC
**37**	4.5896	270.14	0.0120	αHOMO-3 -> αLUMO 0.44882<br>αHOMO -> αLUMO -0.34386<br>βHOMO-17 -> βLUMO -0.38195<br>βHOMO-15 -> βLUMO -0.30879	mbmi(π) -> Fe(d)<br>mbmi(π) -> Fe(d)<br>Fe(d) -> Fe(d)<br>Fe(d)/mbmi(σ)-> Fe(d)	LMCT
**39**	4.6370	267.38	0.0150	βHOMO-1 ->155B 0.34731<br>βHOMO-1 ->157B -0.33431	Fe(d) -> Fe(d)/mbmi(π)<br>Fe(d) -> Fe(d)/mbmi(π)	MLCT/MC
**40**	4.6453	266.90	0.0065	αHOMO-5 -> αLUMO+1 0.24344<br>βHOMO-1 ->156B 0.22637<br>βHOMO-1 ->157B 0.34233	mbmi(π) -> Fe(d)<br>Fe(d) -> mbmi(π)<br>Fe(d) -> Fe(d)/mbmi(π)	MLCT

Table S9. *Relative energies to the singlet ground state at B3LYP*/6-311G(d) for the singlet, triplet and quintet states of* $[Fe(mbmi)_2(bpy)]^{2+}$*. The distances are averaged distances for the four iron-carbene bonds and two iron-bpy bonds.*

**(Fe(II)-C) Distance$_{avg}$ /Å**	$^1[Fe(II)(mbmi)_2(bpy)]^{2+}$/eV	$^3[Fe(II)(mbmi)_2(bpy)]^{2+}$/eV	$^5[Fe(II)(mbmi)_2(bpy)]^{2+}$/eV
**2.04**	0.21	1.31	2.80
**2.05**	0.00	1.53	2.99
**2.16**	0.74	0.83	1.81
**2.28**	1.35	1.45	1.08

Table S10. *Relative energies to the doublet ground state at B3LYP*/6-311G(d) for the doublet, quartet and septet states of* $[Fe(mbmi)_3]^{+3}$*. The distances are averaged distances for the six iron-carbene bonds.*

**(Fe(III)-C) Distance$_{avg}$ /Å**	$^2[Fe(III)(mbmi)_3]^{3+}$/eV	$^4[Fe(II)(mbmi)_3]^{3+}$/eV	$^6[Fe(II)(mbmi)_3]^{3+}$/eV
**2.06**	0.00	2.14	3.08
**2.19**	0.76	1.02	2.11
**2.29**	1.26	1.51	1.52

	$^{3}[Fe(mbmi)_2(bpy)]^{2+}_{MC}$	$^{3}[Fe(mbmi)_2(bpy)]^{2+}_{CT}$	$^{5}[Fe(mbmi)_2(bpy)]^{2+}_{MC}$
**Fe Spin Density**	**2.04**	**1.10**	**3.74**
	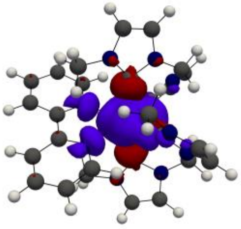	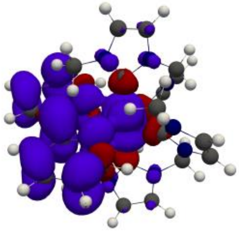	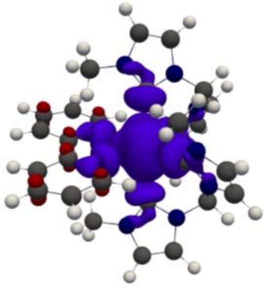
	$^{2}[Fe(mbmi)_3]^{3+}_{GS}$	$^{4}[Fe(mbmi)_3]^{3+}_{MC}$	$^{6}[Fe(mbmi)_3]^{3+}_{MC}$
**Fe Spin Density**	**1.12**	**2.84**	**4.15**
	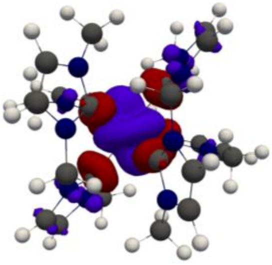	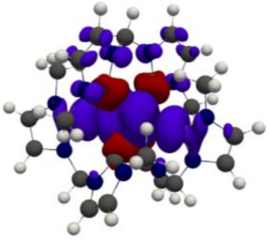	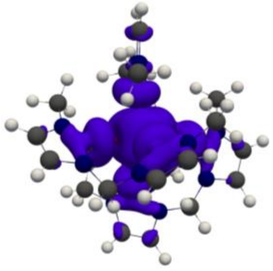

Figure S13. *Spin density plots of optimised triplet metal center and charge transfer states and quintet metal center state in $[Fe(mbmi)_2(bpy)]^{2+}$. The second row contains the spin densities of the optimized doublet, quartet and septet metal center states in $[Fe(mbmi)_3]^{3+}$.*

Table S11. *Cartesian coordinates of the singlet ground state of the $[Fe(mbmi)_2(bpy)]^{2+}$ complex.*

C	1.456640	0.293035	-1.425410
N	2.473546	1.206092	-1.346942
C	2.670100	2.052508	-0.188557

N	1.423013	2.690597	0.187000
C	1.307763	4.051419	0.430854
C	0.021188	4.255371	0.787260
N	-0.612449	3.018604	0.748645
C	0.242423	2.014787	0.373897
N	1.637277	-0.251658	-2.673270
C	2.735578	0.304813	-3.321382
C	3.262496	1.229772	-2.488210
Fe	0.094284	0.000024	0.000048
C	1.456542	-0.293238	1.425496
N	1.637261	0.251480	2.673330
C	2.735447	-0.305167	3.321486
C	3.262184	-1.230286	2.488375
N	2.473248	-1.206518	1.347096
C	-2.024688	2.898642	1.103111
C	0.850994	1.313729	3.299657
C	2.669757	-2.052926	0.188699
N	1.422592	-2.690745	-0.187072
C	0.242128	-2.014730	-0.374020
N	-0.612853	-3.018365	-0.749011
C	0.020620	-4.255210	-0.787797
C	1.307185	-4.051503	-0.431211

C	-2.025069	-2.898173	-1.103499
N	-1.486685	0.474077	-1.211333
C	-2.724438	0.302521	-0.668566
C	-3.793142	1.294983	-2.586812
C	-2.526254	1.502080	-3.128745
C	-1.413706	1.084366	-2.412295
C	-3.887375	-0.694881	1.338812
C	-2.724438	-0.302310	0.668749
C	-3.793164	-1.294712	2.587016
C	-2.526278	-1.501859	3.128931
C	-1.413720	-1.084206	2.412460
N	-1.486681	-0.473923	1.211495
H	3.397447	-2.824436	0.435102
H	3.056393	-1.463961	-0.644667
H	-0.165987	1.296751	2.921062
H	0.827601	1.143359	4.376929
H	1.295902	2.292601	3.104819
H	-2.655477	-2.905635	-0.212949
H	-2.196925	-1.987105	-1.669699
H	-2.296742	-3.747044	-1.731166
H	-2.655078	2.906137	0.212549
H	-2.196674	1.987632	1.669366

H	-2.296254	3.747589	1.730723
H	3.397988	2.823849	-0.434900
H	3.056497	1.463502	0.644888
C	0.850858	-1.313751	-3.299668
H	0.827717	-1.143437	-4.376954
H	1.295480	-2.292726	-3.104696
H	-0.166197	-1.296494	-2.921284
C	-3.887365	0.695146	-1.338612
H	-4.858949	-0.549303	0.881673
H	-2.392010	-1.981489	4.092770
H	-0.418361	-1.243923	2.806293
H	2.137014	-4.733948	-0.333979
H	2.137695	4.733733	0.333576
H	-0.501258	5.158413	1.061500
H	4.106163	1.892624	-2.601038
H	3.030802	-0.001514	-4.312733
H	3.030709	0.001145	4.312830
H	4.105713	-1.893305	2.601253
H	-0.501923	-5.158130	-1.062250
H	-0.418341	1.244041	-2.806130
H	-2.391980	1.981715	-4.092581
H	-4.687436	1.605833	-3.117614

H	-4.858940	0.549615	-0.881461
H	-4.687465	-1.605515	3.117833

Table S12. *Cartesian coordinates of the triplet metal-to-ligand charge-transfer state of the [Fe(mbmi)$_2$(bpy)]$^{2+}$ complex.*

C	1.492078	0.345500	-1.411181
N	2.487221	1.265880	-1.288321
C	2.663233	2.086966	-0.106627
N	1.402939	2.701502	0.274965
C	1.269439	4.051584	0.547462
C	-0.024748	4.233070	0.898583
N	-0.646129	2.996713	0.824384
C	0.224976	2.024116	0.436201
N	1.670680	-0.157408	-2.666888
C	2.760514	0.436386	-3.293016
C	3.274064	1.340396	-2.428073
Fe	0.080252	0.000240	0.000058
C	1.491643	-0.347001	1.411086
N	1.671129	0.156121	2.666575
C	2.760214	-0.438981	3.292767
C	3.272425	-1.343949	2.428020

N	2.485485	-1.268766	1.288378
C	-2.067632	2.852069	1.142698
C	0.879432	1.191161	3.337241
C	2.660593	-2.089954	0.106630
N	1.399597	-2.702796	-0.275323
C	0.222607	-2.023868	-0.437263
N	-0.649492	-2.995373	-0.826011
C	-0.029649	-4.232538	-0.899808
C	1.264525	-4.052721	-0.547847
C	-2.070635	-2.849102	-1.145188
N	-1.459099	0.501808	-1.205121
C	-2.722459	0.288808	-0.644798
C	-3.779248	1.289583	-2.587598
C	-2.488103	1.562405	-3.112759
C	-1.388662	1.159042	-2.388121
C	-3.886134	-0.665608	1.371915
C	-2.722298	-0.286593	0.646527
C	-3.778801	-1.286911	2.589720
C	-2.487567	-1.560946	3.114039
C	-1.388212	-1.158300	2.388894
N	-1.458741	-0.500736	1.206066
H	3.377606	-2.877210	0.329287

H	3.050263	-1.487361	-0.714294
H	-0.139140	1.179218	2.962132
H	0.861447	0.974317	4.405420
H	1.323346	2.176568	3.181369
H	-2.667705	-2.842854	-0.232899
H	-2.238701	-1.929429	-1.697746
H	-2.368306	-3.694618	-1.764505
H	-2.664123	2.846113	0.230026
H	-2.237029	1.932764	1.695497
H	-2.364812	3.698119	1.761523
H	3.381321	2.873251	-0.329277
H	3.051991	1.483976	0.714445
C	0.877697	-1.191278	-3.337838
H	0.860456	-0.974372	-4.406014
H	1.320128	-2.177331	-3.181846
H	-0.141005	-1.177865	-2.963144
C	-3.886400	0.668662	-1.369574
H	-4.865091	-0.473924	0.945392
H	-2.352570	-2.073612	4.060098
H	-0.391108	-1.363906	2.760029
H	2.085669	-4.747944	-0.471371
H	2.091514	4.745764	0.471528

H	-0.558008	5.124262	1.189403
H	4.105881	2.021223	-2.516843
H	3.060276	0.167936	-4.293660
H	3.060463	-0.170656	4.293301
H	4.103327	-2.025880	2.516885
H	-0.563843	-5.123038	-1.191032
H	-0.391559	1.363741	-2.759802
H	-2.353241	2.074706	-4.059035
H	-4.670864	1.581666	-3.135170
H	-4.865272	0.477897	-0.942446
H	-4.670338	-1.578375	3.137750

Table S13. *Cartesian coordinates of the triplet metal-centred state of the [Fe(mbmi)$_2$(bpy)]$^{2+}$ complex.*

C	1.481774	1.151433	-1.269598
N	2.512517	1.872146	-0.741678
C	2.762814	1.926197	0.690476
N	1.550825	2.266041	1.407813
C	1.444598	3.327085	2.296581
C	0.183733	3.282078	2.779947
N	-0.446095	2.200505	2.172685
C	0.382849	1.549929	1.302185

N	1.609173	1.358201	-2.609861
C	2.691681	2.179621	-2.900378
C	3.267383	2.505611	-1.717835
Fe	0.228955	0.000018	0.000133
C	1.484690	-1.148452	1.269845
N	1.612317	-1.355213	2.610094
C	2.696567	-2.174332	2.900613
C	3.273187	-2.498802	1.718093
N	2.517094	-1.866778	0.741953
C	-1.829083	1.858464	2.489373
C	0.759709	-0.782851	3.649567
C	2.767761	-1.919922	-0.690181
N	1.556693	-2.262482	-1.407789
C	0.386910	-1.549380	-1.301954
N	-0.440463	-2.201942	-2.172480
C	0.192113	-3.281747	-2.780028
C	1.453129	-3.323608	-2.296780
C	-1.824449	-1.863573	-2.488775
N	-1.700929	1.105458	-0.738698
C	-2.913280	0.647565	-0.355726
C	-4.005590	2.593276	-1.263673
C	-2.753777	3.067214	-1.649421

C	-1.636465	2.292305	-1.359300
C	-4.082153	-1.380371	0.604412
C	-2.911785	-0.653121	0.355215
C	-3.999850	-2.600987	1.263695
C	-2.746919	-3.072502	1.648776
C	-1.631263	-2.295442	1.358083
N	-1.698322	-1.108650	0.737593
H	3.525064	-2.675979	-0.888827
H	3.138406	-0.952096	-1.034929
H	-0.179396	-0.464516	3.207091
H	0.557601	-1.539570	4.409249
H	1.251249	0.073862	4.116815
H	-2.517127	-2.462474	-1.893414
H	-1.995460	-0.808968	-2.297841
H	-2.003881	-2.061244	-3.546717
H	-2.523523	2.455765	1.894458
H	-1.997422	0.803493	2.298147
H	-2.008642	2.055316	3.547448
H	3.518311	2.684072	0.889088
H	3.135648	0.959323	1.035529
C	0.757654	0.784277	-3.649353
H	0.551663	1.541459	-4.407546

H	1.252059	-0.069702	-4.118581
H	-0.179619	0.461459	-3.206270
C	-4.085218	1.372501	-0.604348
H	-5.045924	-1.012338	0.272443
H	-2.629410	-4.022904	2.159071
H	-0.635459	-2.633281	1.627845
H	2.274036	-3.992372	-2.503864
H	2.263799	3.998000	2.503447
H	-0.321752	3.911186	3.495982
H	4.124762	3.119060	-1.488266
H	2.943991	2.464105	-3.910249
H	2.949319	-2.458521	3.910457
H	4.131904	-3.110379	1.488536
H	-0.311806	-3.912011	-3.496143
H	-0.641460	2.632019	-1.629675
H	-2.638401	4.017831	-2.159804
H	-4.905083	3.168176	-1.462289
H	-5.048126	1.002611	-0.271922
H	-4.898129	-3.177622	1.462768

Table S14. *Cartesian coordinates of the quintet metal-centered state of the [Fe(mbmi)$_2$(bpy)]$^{2+}$ complex.*

C	1.620357	-1.039213	1.372442
N	2.524872	-1.928656	0.868876
C	2.659677	-2.188982	-0.559175
N	1.431043	-2.707599	-1.135768
C	1.292303	-3.973445	-1.689749
C	0.017111	-4.042892	-2.141112
N	-0.578392	-2.823242	-1.842805
C	0.276650	-1.975027	-1.209667
N	1.854255	-1.079706	2.711911
C	2.873146	-1.967783	3.031306
C	3.299457	-2.507823	1.862999
Fe	0.161158	0.000013	-0.000022
C	1.620191	1.039480	-1.372441
N	1.854103	1.080010	-2.711905
C	2.872904	1.968198	-3.031275
C	3.299079	2.508338	-1.862964
N	2.524557	1.929063	-0.868857
C	-1.965610	-2.523237	-2.186620
C	1.135197	0.301641	-3.719069
C	2.659302	2.189399	0.559200
N	1.430580	2.707806	1.135792
C	0.276310	1.975039	1.209693

N	-0.578877	2.823114	1.842822
C	0.016432	4.042852	2.141162
C	1.291633	3.973622	1.689793
C	-1.966055	2.522887	2.186604
N	-1.687073	-0.907401	0.982278
C	-2.900300	-0.539924	0.507733
C	-3.999249	-2.171391	1.901206
C	-2.748255	-2.549439	2.383157
C	-1.626696	-1.893488	1.890475
C	-4.075137	1.159419	-0.951249
C	-2.900380	0.539499	-0.507758
C	-3.999572	2.170800	-1.901232
C	-2.748635	2.549027	-2.383191
C	-1.626979	1.893242	-1.890509
N	-1.687208	0.907150	-0.982307
H	3.455410	2.916289	0.708771
H	2.925828	1.262027	1.065306
H	0.426517	-0.349872	-3.213440
H	0.603601	0.968673	-4.401584
H	1.839724	-0.307633	-4.289431
H	-2.648663	2.965644	1.457294
H	-2.101412	1.444838	2.208150

H	-2.188865	2.927603	3.175336
H	-2.648162	-2.966090	-1.457317
H	-2.101132	-1.445209	-2.208185
H	-2.188336	-2.928001	-3.175351
H	3.455904	-2.915744	-0.708730
H	2.926059	-1.261573	-1.065290
C	1.135244	-0.301417	3.719062
H	0.603741	-0.968511	4.401587
H	1.839686	0.307965	4.289414
H	0.426475	0.349990	3.213422
C	-4.074965	-1.160017	0.951227
H	-5.040214	0.870556	-0.552343
H	-2.637476	3.336933	-3.120823
H	-0.632316	2.167339	-2.226963
H	2.097389	4.690708	1.724405
H	2.098177	-4.690399	-1.724354
H	-0.510479	-4.836656	-2.647407
H	4.072761	-3.231615	1.656514
H	3.198024	-2.134044	4.046928
H	3.197799	2.134483	-4.046888
H	4.072268	3.232251	-1.656466
H	-0.511287	4.836521	2.647471

H	-0.631992	-2.167439	2.226924
H	-2.636978	-3.337332	3.120786
H	-4.902825	-2.660669	2.252077
H	-5.040085	-0.871295	0.552326
H	-4.903221	2.659945	-2.252102

Table S15. *Cartesian coordinates of the doublet ground state of the $[Fe(mbmi)_3]^{3+}$ complex.*

C	-0.486751	-1.624808	-1.085016
N	-0.677041	-2.853699	-0.529919
C	-0.491705	-3.142458	0.880894
N	0.730003	-2.530224	1.382101
C	1.627182	-3.212941	2.187730
C	2.577708	-2.315244	2.534293
N	2.242845	-1.113528	1.931452
C	1.094317	-1.219700	1.207717
N	-0.747845	-1.822704	-2.408648
C	-1.101686	-3.143886	-2.656549
C	-1.053363	-3.798533	-1.473976
Fe	0.020169	0.039984	-0.022131
C	-1.564551	-0.317388	1.213852
N	-1.657201	-0.468228	2.566142

C	-2.962060	-0.754267	2.949558
C	-3.713351	-0.773612	1.825298
N	-2.841892	-0.504688	0.779497
C	3.077701	0.074907	2.110230
C	-0.594499	-0.295470	3.561811
C	-3.245808	-0.418199	-0.612297
N	-2.637682	0.734044	-1.259457
C	-1.305849	1.055189	-1.222148
N	-1.219401	2.133414	-2.047518
C	-2.453823	2.470053	-2.579045
C	-3.353015	1.590456	-2.081023
C	-0.015799	2.888466	-2.386565
C	1.863722	0.193783	-0.971104
N	2.721581	1.258976	-0.911499
C	3.931119	1.005369	-1.543310
C	3.854030	-0.260316	-1.997263
N	2.612117	-0.752862	-1.634986
C	2.562171	2.563457	-0.301873
N	1.458864	2.696788	0.632096
C	1.367560	3.904847	1.311450
C	0.152025	3.915225	1.891300
N	-0.474803	2.726838	1.554314

C	0.335605	1.926809	0.782727
C	2.319404	-2.164780	-1.907632
C	-1.879506	2.551105	1.941338
H	4.720295	1.738627	-1.600413
H	2.160740	4.635849	1.302096
H	-4.766736	-0.949863	1.674847
H	-4.416157	1.494801	-2.235117
H	-2.418079	2.000027	1.179307
H	-1.967935	2.054607	2.906597
H	-2.325012	3.542242	2.018684
H	-0.327785	4.658690	2.507627
H	2.442350	3.312695	-1.089595
H	3.484855	2.793125	0.235380
H	4.572410	-0.858971	-2.533717
H	1.711932	-2.278803	-2.803427
H	3.269280	-2.672359	-2.069011
H	1.832226	-2.627646	-1.056409
H	-4.326662	-0.300869	-0.651974
H	-2.980844	-1.333440	-1.142372
H	-3.235014	-0.903502	3.982171
H	0.153953	0.397584	3.188356
H	-1.034011	0.120464	4.468788

H	-0.129173	-1.252187	3.805767
H	-2.578681	3.299312	-3.256985
H	0.142279	3.694819	-1.668383
H	0.839659	2.219042	-2.412841
H	-0.144062	3.321339	-3.378047
H	3.843489	0.123106	1.334335
H	2.460580	0.967542	2.090273
H	3.564159	0.011404	3.083060
H	-0.408865	-4.220181	1.004267
H	-1.351461	-2.797068	1.455277
C	-0.623501	-0.849800	-3.499268
H	-0.343874	-1.384206	-4.407254
H	-1.570070	-0.335712	-3.674968

Table S16. *Cartesian coordinates of the quartet metal-centered state of the $[Fe(mbmi)_3]^{3+}$ complex.*

C	0.571883	-1.472516	-1.242203
N	1.318958	-2.532044	-0.810582
C	1.808738	-2.722230	0.551367
N	2.524237	-1.553899	1.039635
C	3.800814	-1.594319	1.590477
C	4.078861	-0.326863	1.978584
N	2.970962	0.445600	1.650602

C	1.994750	-0.295416	1.073141
N	0.393480	-1.712081	-2.568181
C	1.001800	-2.899444	-2.949337
C	1.595398	-3.412547	-1.846087
Fe	-0.039302	0.126311	-0.074698
C	-0.796517	-1.225470	1.340015
N	-0.578926	-1.349230	2.678560
C	-1.287174	-2.419848	3.206047
C	-1.979507	-2.978568	2.186535
N	-1.665948	-2.239922	1.055394
C	2.889403	1.875933	1.943518
C	0.240445	-0.486102	3.534866
C	-2.217408	-2.525503	-0.264206
N	-2.841224	-1.354157	-0.851202
C	-2.180473	-0.184678	-1.098776
N	-3.123064	0.582175	-1.705428
C	-4.333229	-0.088524	-1.831331
C	-4.159062	-1.316383	-1.289302
C	-2.943000	1.956097	-2.169100
C	1.224096	1.430742	-1.141424
N	1.116270	2.792111	-1.099866
C	2.174225	3.423070	-1.728732

C	2.985961	2.436343	-2.168583
N	2.408238	1.234897	-1.802524
C	-0.016677	3.562615	-0.635335
N	-0.616604	3.062174	0.591679
C	-1.287061	3.947728	1.418957
C	-2.087042	3.195293	2.205894
N	-1.897136	1.874027	1.840611
C	-0.963055	1.760908	0.848385
C	3.091873	-0.019918	-2.130934
C	-2.760683	0.839516	2.421571
H	2.251418	4.496112	-1.803323
H	-1.147947	5.015653	1.360656
H	-2.654673	-3.819335	2.162393
H	-4.833138	-2.152385	-1.186454
H	-2.872291	0.015363	1.726311
H	-2.366060	0.487696	3.374122
H	-3.742508	1.281766	2.589825
H	-2.774375	3.478985	2.987019
H	-0.779460	3.597475	-1.415922
H	0.325312	4.579021	-0.447561
H	3.925475	2.482388	-2.695671
H	2.630991	-0.497021	-2.994724

H	4.126116	0.216699	-2.375015
H	3.086285	-0.691990	-1.279982
H	-2.970436	-3.302801	-0.154675
H	-1.432497	-2.897930	-0.920713
H	-1.241821	-2.677652	4.252511
H	0.340977	0.496043	3.082926
H	-0.254990	-0.385132	4.501184
H	1.227582	-0.924146	3.689749
H	-5.197723	0.362731	-2.292658
H	-3.130457	2.663470	-1.358532
H	-1.935736	2.083649	-2.560974
H	-3.649148	2.152796	-2.975681
H	3.664384	2.415617	1.395879
H	1.911311	2.244216	1.645288
H	3.022727	2.041205	3.014450
H	2.490949	-3.569449	0.548260
H	0.976904	-2.956731	1.213563
C	-0.301503	-0.853944	-3.532152
H	0.192930	-0.950911	-4.499055
H	-1.345930	-1.152653	-3.630842

Table S17. *Cartesian coordinates of the hextet metal-centered state of the [Fe(mbmi)$_3$]$^{3+}$ complex.*

C	0.291734	-1.840092	-1.194092
N	0.708240	-2.975283	-0.562601
C	0.856665	-3.097598	0.883212
N	1.730986	-2.070638	1.433091
C	2.836624	-2.344182	2.228918
C	3.339448	-1.142503	2.599547
N	2.533653	-0.172835	2.018512
C	1.530577	-0.726253	1.294408
N	0.276245	-2.181630	-2.507112
C	0.665982	-3.498882	-2.690636
C	0.944602	-4.003024	-1.462054
Fe	-0.050355	0.100389	-0.140833
C	-1.414224	-0.882087	1.426239
N	-1.430632	-0.899942	2.785675
C	-2.499955	-1.636292	3.271391
C	-3.184181	-2.095658	2.195001
N	-2.505717	-1.625711	1.081621
C	2.769348	1.258553	2.208138
C	-0.473853	-0.241320	3.677747
C	-2.883923	-1.924595	-0.293742
N	-3.038305	-0.713926	-1.080965

C	-2.036107	0.185095	-1.295752
N	-2.594635	1.094266	-2.134068
C	-3.911595	0.773875	-2.429318
C	-4.196732	-0.370246	-1.762015
C	-1.931692	2.280532	-2.672548
C	1.878318	0.997719	-1.086564
N	2.145339	2.337072	-1.099974
C	3.463057	2.603909	-1.439641
C	4.046662	1.399626	-1.639760
N	3.075699	0.436500	-1.418222
C	1.158487	3.395734	-1.005721
N	0.287312	3.312522	0.160714
C	-0.115786	4.463829	0.820103
C	-1.169571	4.109195	1.591143
N	-1.390433	2.759084	1.376323
C	-0.488578	2.234781	0.500724
C	3.369096	-0.987780	-1.595351
C	-2.546634	2.085348	1.972333
H	3.857088	3.605763	-1.508171
H	0.367536	5.417228	0.675118
H	-4.074032	-2.701410	2.125795
H	-5.096894	-0.962833	-1.718085

H	-2.785482	1.197488	1.396435
H	-2.349595	1.821962	3.012257
H	-3.396719	2.767148	1.935732
H	-1.779212	4.691284	2.264506
H	0.546310	3.398122	-1.908231
H	1.689840	4.343639	-0.954359
H	5.058007	1.144185	-1.913959
H	2.875641	-1.370563	-2.488601
H	4.445604	-1.102008	-1.712292
H	3.052625	-1.554377	-0.724289
H	-3.830262	-2.460883	-0.285794
H	-2.126210	-2.560015	-0.751241
H	-2.683920	-1.756923	4.327519
H	-0.029575	0.612940	3.174503
H	-1.001489	0.105435	4.566575
H	0.308851	-0.939406	3.980894
H	-4.519851	1.379653	-3.082334
H	-1.940095	3.088159	-1.938229
H	-0.909627	2.032741	-2.955844
H	-2.465687	2.607754	-3.564059
H	3.693142	1.556623	1.708330
H	1.935780	1.818676	1.794006

H	2.850403	1.474662	3.274766
H	1.288181	-4.071088	1.104532
H	-0.122130	-3.035554	1.356887
C	-0.111053	-1.301424	-3.612947
H	0.379493	-1.645184	-4.523278
H	-1.192594	-1.323859	-3.756640

S13 References

S1. Benz, M.; Spingler, B.; Alberto, R.; Braband, H. Toward Organometallic 99mTc Imaging Agents:Synthesis of Water-Stable 99Tc–NHC Complexes. *J. Am. Chem. Soc.* **2013**, *135* (46), 17566-17572. DOI: 10.1021/ja409499u.

S2. Charron, F. F.; Reiff, W. M. The second isomer of dichloro(2,2'-bipyridine)iron(II): syntheses and spectroscopic and magnetic characterizations of three related dichloro(.alpha.-diimine)iron(II) complexes containing five-coordinate, high-spin iron(II). *Inorg. Chem.* **1986**, *25* (16), 2786-2790. DOI: 10.1021/ic00236a026.

S3. CrysAlis PRO. Agilent Technologies **2013**. www.agilent.com/cs/library/usermanuals/Public/CrysAlis_Pro_User_Manual.pdf.

S4. Sheldrick, G. M. Crystal structure refinement with *SHELXL*. *Acta Cryst.* **2015**, *C71*, 3-8. DOI: 10.1107/S2053229614024218.

S5. Sheldrick, G. M. A Short History of *SHELXL Acta Cryst.* **2008**, *A64*, 112-122. DOI: 10.1107/S0108767307043930.

S6. Dolomanov, O. V.; Bourhis, L. J.; Gildea, R. J.; Howard, J. A. K.; Puschmann, H. *OLEX2*: a complete structure solution, refinement and analysis program. *J. Appl. Cryst.* **2009**, *42*, 339-341. DOI: 10.1107/S0021889808042726.

S7. Liu, Y.; Kjær, K. S.; Fredin, L. A.; Chábera, P.; Harlang, T.; Canton, S. E.; Lidin, S.; Zhang, J.; Lomoth, R.; Bergquist, K.-E.; Persson, P.; Wärnmark, K.; Sundström, V. A Heteroleptic Ferrous Complex with Mesoionic Bis(1,2,3-triazol-5-ylidene) Ligands:

Taming the MLCT Excited State of Iron(II). *Chem. Eur. J.* **2015**, *21* (9), 3628-3639. DOI: 10.1002/chem.201405184.

S8. Chábera, P.; Liu, Y.; Prakash, O.; Thyrhaug, E.; Nahhas, A. E.; Honarfar, A.; Essén, S.; Fredin, L. A.; Harlang, T. C. B.; Kjær, K. S.; Handrup, K.; Ericson, F.; Tatsuno, H.; Morgan, K.; Schnadt, J.; Häggström, L.; Ericsson, T.; Sobkowiak, A.; Lidin, S.; Huang, P.; Styring, S.; Uhlig, J.; Bendix, J.; Lomoth, R.; Sundström, V.; Persson, P.; Wärnmark, K. A low-spin Fe(iii) complex with 100-ps ligand-to-metal charge transfer photoluminescence. *Nature* **2017**, *543* (7647), 695-699. DOI: 10.1038/nature21430.

S9. Kjær, K. S.; Kaul, N.; Prakash, O.; Chábera, P.; Rosemann, N. W.; Honarfar, A.; Gordivska, O.; Fredin, L. A.; Bergquist, K.-E.; Häggström, L.; Ericsson, T.; Lindh, L.; Yartsev, A.; Styring, S.; Huang, P.; Uhlig, J.; Bendix, J.; Strand, D.; Sundström, V.; Persson, P.; Lomoth, R.; Wärnmark, K. Luminescence and reactivity of a charge-transfer excited iron complex with nanosecond lifetime. *Science* **2019**, *363* (6424), 249-253. DOI: doi:10.1126/science.aau7160.

S10. Prakash, O.; Lindh, L.; Kaul, N.; Rosemann, N. W.; Losada, I. B.; Johnson, C.; Chábera, P.; Ilic, A.; Schwarz, J.; Gupta, A. K.; Uhlig, J.; Ericsson, T.; Häggström, L.; Huang, P.; Bendix, J.; Strand, D.; Yartsev, A.; Lomoth, R.; Persson, P.; Wärnmark, K. Photophysical Integrity of the Iron(III) Scorpionate Framework in Iron(III)–NHC Complexes with Long-Lived 2LMCT Excited States. *Inorg. Chem.* **2022**, *61* (44), 17515-17526. DOI: 10.1021/acs.inorgchem.2c02410.

S11. Frisch, M. J.; Trucks, G. W.; Schlegel, H. B.; Scuseria, G. E.; Robb, M. A.; Cheeseman, J. R.; Scalmani, G.; Barone, V.; Mennucci, B.; Petersson, G. A.; Nakatsuji, H.; Caricato, M.; Li, X.; Hratchian, H. P.; Izmaylov, A. F.; Bloino, J.; Zheng, G.; Sonnenberg, J. L.; Hada, M.; Ehara, M.; Toyota, K.; Fukuda, R.; Hasegawa, J.; Ishida, M.; Nakajima, T.; Honda, Y.; Kitao, O.; Nakai, H.; Vreven, T.; Montgomery, J. A. J.; Peralta, J. E.; Ogliaro, F.; Bearpark, M.; Heyd, J. J.; Brothers, E.; Kudin, K. N.; Staroverov, V. N.; Kobayashi, R.; Normand, J.; Raghavachari, K.; Rendell, A.; Burant, J. C.; Iyengar, S. S.; Tomasi, J.; Cossi, M.; Rega, N.; Millam, J. M.; Klene, M.; Knox, J. E.; Cross, J. B.; Bakken, V.; Adamo, C.; Jaramillo, J.; Gomperts, R.; Stratmann, R. E.; Yazyev, O.; Austin, A. J.; Cammi, R.; Pomelli, C.; Ochterski, J. W.; Martin, R. L.; Morokuma, K.; Zakrzewski, V. G.; Voth, G. A.; Salvador, P.; Dannenberg, J. J.; Dapprich, S.; Daniels, A. D.; Farkas, O.; Foresman, J.

B.; Ortiz, J. V.; Cioslowski, J.; Fox, D. J. Gaussian 09, Revision **C.01**. Gaussian, Inc., Wallingford CT, **2009**. https://gaussian.com/g09_c01/.
